# Large-Scale Synthesis (75 g/Batch) of Single-Atom Catalysts for Selective Electrochemical CO_2_ Reduction to CO and Commercialization Potential Analysis

**DOI:** 10.1021/acsomega.6c02693

**Published:** 2026-06-02

**Authors:** Carter Racine, Ahmed Badreldin, John Pellessier, Yayun Chen, Shaoqin Chen, Shengyao Wang, Jin Feng, Chengcheng Fei, Yun Hang Hu, Ying Li

**Affiliations:** † J. Mike Walker ’66 Department of Mechanical Engineering, 14736 Texas A&M University, College Station, Texas 77843, United States; ‡ Department of Chemical Engineering, 8083 University of Mississippi, Oxford, Mississippi 38677, United States; § Department of Agriculture Economics, Texas A&M University, College Station, Texas 77843, United States; ∥ Department of Materials Science and Engineering, 3968 Michigan Technological University, Houghton, Michigan 49931, United States; ⊥ Artie McFerrin Department of Chemical Engineering, Texas A&M University, College Station, Texas 77843, United States

## Abstract

A promising approach to mitigate climate change is to use renewable electricity to convert carbon dioxide (CO_2_) emissions into useful products like carbon monoxide (CO) and hydrocarbons. While research has largely focused on developing high-performance catalysts for electrochemical CO_2_ reduction (eCO_2_R), scalability of catalyst synthesis remains underexplored. Metal–nitrogen–carbon (M–N–C) catalysts with dominant single-atom sites are among the most effective materials for CO production, but conventional synthesis methods rely on energy-intensive steps and complex pre- and post-treatment processes, hindering scalability and adding negative environmental impacts. This work demonstrates a scalable, single-step synthesis of M–N–C (M = Ni/Fe) catalysts using commercially available multiwalled carbon nanotubes (MWCNTs) and melamine as the feedstock. The method leverages inherent Ni/Fe impurities in MWCNTs, requiring only moderate-temperature pyrolysis (650 °C) without any pre- or post-treatments. Batch sizes up to 75 g were successfully produced using both regular-grade (>95% carbon content) and cheaper but more impure industrial-grade (>90% carbon content) MWCNTs. Structural, chemical, and electrochemical analyses confirmed reproducibility. Catalysts consistently achieved >98% CO selectivity at commercially relevant current densities (500 mA cm^–2^), outperforming benchmark Ag nanoparticle catalysts. Techno-economic analysis (TEA) further underscored the commercialization potential, yielding a base case minimum selling price (MSP) of $145 per tonne of CO, $255 below the current CO market price and lower than that obtained using Ag catalysts. Life cycle assessment (LCA) showed significant environmental benefits, registering 21% lower CO_2_-equivalent emissions compared to using Ag catalysts. Overall, this scalable approach produces high-performance M–N–C catalysts while delivering clear environmental and economic advantages, advancing the commercialization of eCO_2_R technology.

## Graphical abstract

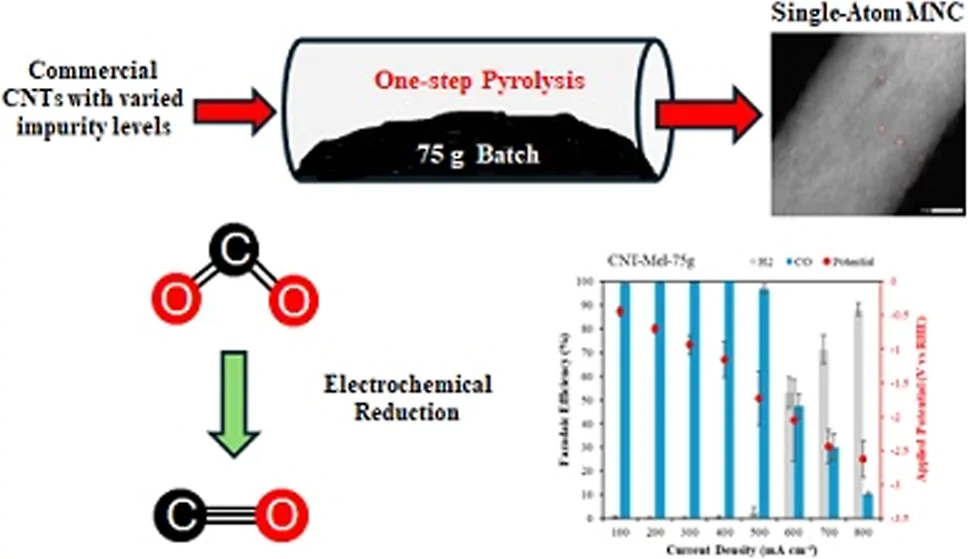

## 1 Introduction

Carbon capture and utilization (CCU) is a promising pathway for commercializing the removal of anthropogenic CO_2_ into higher-value commodities,^1^ specifically decreasing the usage of virgin fossil fuels in carbon-intensive industries.^2^ Electrochemical CO_2_ reduction (eCO_2_R) represents a compelling approach within CCU, as it enables the use of renewable electricity to decarbonize hard-to-electrify sectors such as chemicals, paper, aviation, and maritime transport.^3^ The commercialization of eCO_2_R is reliant on the scalable production of high-performing electrocatalysts that consistently and durably attain high product selectivity at industrially relevant current densities (>300 mA cm^–2^ according to Kibria et al.).^4,5^ Previous techno-economic analyses (TEA) have identified CO_2_ to CO conversion as one of the most commercially viable pathways for upscaling the utilization of eCO_2_R, based on thoroughly demonstrated high selectivity (>90% Faradaic efficiency of CO production) and low overpotentials (roughly 0.5 V) at intermediate current densities (>100 mA cm^–2^).^5,6^ Notwithstanding, ongoing work continues to be generated on improving the aforementioned electrocatalytic performance metrics, although a clear lack of cost-effective and scalable production methods for these catalysts is expected to hinder their commercial viability in eCO_2_R.^7^

The most widely researched catalysts for eCO_2_R to CO are noble metal-based (Au or Ag) nanoparticles; however, their scalable production is not feasible due to their high cost.^8^ Equivalent, and even greater, performance has been demonstrated in catalysts made of non-noble metals and nonmetallic materials. Notwithstanding, the intricacies of their synthesis methods hold back their scalability.^9–12^ M–N–C-based single atom catalysts (SACs) have to that end garnered much attention, even outperforming Ag nanoparticles in some cases.^13–15^ Briefly, M–N–C catalysts employ carbon-based substrates whereby single-atom metal sites are coordinated with nonmetallic nitrogen or other nonmetallic heteroatoms (i.e., S and P).^16^ These catalysts have an advantage in that they mostly employ earth-abundant transition metals such as Fe, Ni, Co, Zn, and Cu, which are inexpensive relative to traditional Au- and Ag-based materials.^17^

Although most transition metals can be used to make M–N–C SACs when focusing on CO production, Ni and Fe are the most commonly studied and some of the most promising.^18^ Wang et al. report Ni-based M–N–Cs as superior in both partial current density and Faradaic efficiency to other transition metals, Fe being the next best, followed by Cu and Co.^19^ Other researchers have also found this order of selectivity to be the case; however, the selectivity differences between Ni and Fe are not egregious. Ni and Fe have also shown the highest thermodynamic favorability toward eCO_2_R to CO.^19,20^ One study compared the selectivity of transition-metal-centered M–N–Cs and found that Ni centers have the highest peak selectivity of roughly 90% FE CO, while the Fe-centered sample had the second highest peak at about 80% FE CO, although the Fe samples showed lower overpotentials.^21^ Both Ni- and Fe-centered catalysts can be viable depending on the targeted application. Fe-based catalysts are preferred when minimizing the overpotential is critical, whereas Ni-based catalysts are more suitable for maximizing selectivity. Part of this advantage in performance comes from the optimal *COOH binding energy exhibited by Fe and Ni M–N–C’s. This allows for favorable CO_2_-to-CO conversion dynamics without excessive CO binding energies, which would lead to blocked active sites that are then unable to perform eCO_2_R and are forced to produce H_2_.^19^ Ni–N–Cs have weak *H, *COOH, and *CO bonding, making competing HER thermodynamically unfavorable compared to other transition metal M–N–Cs such as Co–N–C which has a high binding energy toward *H.^22^ Fe and Ni M–N–Cs also allow for nearly 100% atom utilization, maximizing per metal atom activity compared to nanoparticles.^23^ Full metal atom utilization allows for more cost savings as none of the expensive metal atoms go to waste, contrary to nanoparticles, where only the surface atoms are active, leading to substantial metal atom waste.

The majority of M–N–C synthesis approaches rely on similar steps involving preparation and pretreatment of the carbon substrate, a physiochemical or wet chemical coupling of the metal and nitrogen precursors with the carbon substrate, followed by high-temperature pyrolysis or annealing steps that may be in excess of 1000 °C and 6 h on-stream.^22,24–27^ Prior to catalyst utilization, the resultant product is typically acid washed to remove unreacted or loosely coupled metal nanoparticles.^28^ After this sometimes multiday synthesis process, the overwhelming majority of M–N–C reports only yield tens to a couple hundred milligrams of catalyst per batch. Therefore, a simplified revamp of these synthesis protocols is greatly needed to address both the number of steps and required reagents and solvents needed. This should come without undermining catalytic performance metrics or control over the quality of the produced M–N–C.

Few works have focused on producing practically large batches (gram scale) of transition metal M–N–C SACs for any application, let alone for eCO_2_R to CO. Therefore, some of the following discussions are not explicitly targeting eCO_2_R; however, the issues regarding their scaled-up synthesis still apply. Efforts directed toward addressing the production of commercially meaningful large batches of transition metal M–N–Cs, which have been presented in the literature, have procedures that lead to wasted solvents, precursors, and electrical energy, which all limit the commercial viability of these materials. Zheng et al.^29^ were able to reach ∼1 g per batch for their Ni-SAC by using an ion adsorption method to combine Ni­(NO_3_)_2_·6H_2_O with carbon black and then mixed in a mass ratio of 1:10 with urea before a pyrolysis treatment at 800 °C for 1 h, which sustained 99% FE toward CO at 100 mA cm^–2^ of current density. However, the excessive need for urea hinders the scalability of this catalyst, as larger batches at this ratio will require impractically large amounts of urea and energy-demanding pyrolysis systems to sustain 800 °C. Zhang et al.^30^ was also able to reach ∼1 g per batch scale using a similar adsorption-pyrolysis method when making their Co-SAC. Both works show promise for large-scale synthesis; however, they have only reached 1 g per batch, and directly scaling these methods will lead to exponential increases in material and energy requirements. Liu et al.^31^ synthesized a Co-SAC using 1.32 g of CoCl_2_·6H_2_O, 60 g of urea, and 6 g of PVP, yielding a final batch of only 4 g. Due to the incredibly low yield of viable catalyst material of this synthesis method, larger pyrolysis chambers are required, leading to wasted energy and limited space for further scale-up studies. Yang et al.^32^ was able to produce a Ni-SAC at batch sizes of 1.6 kg with high yield (2.01 kg of precursor materials) using only a few stirring steps and a moderate-temperature pyrolysis. This level of scale and yield is unprecedented; however, eCO_2_R testing was only done in an H-cell, achieving high selectivity (>98% FE CO) at an industrially irrelevant current density of roughly 8 mA cm^–2^. Lyu et al.^22^ generated 75 g of an M–N–C catalyst through a direct physicochemical ball-milling protocol. Briefly, a metal precursor, a nitrogen precursor (pipemidic acid, aminoantipyrine, or other), and high-surface-area fumed silica as a carbon substrate are ball-milled together in a 1:10:4 mass ratio, respectively. Following this, short-term (60–90 min), high-temperature pyrolysis is done at 950–1000 °C under a 500 sccm flow of nitrogen. This is followed by two separate acid washings (HF and HNO_3_) with neutralization and drying in between. A maximum FE CO of 80% was reported at roughly −0.5 V (vs RHE) and 25 mA cm^–2^ current density in a flow reactor; testing done at industrially relevant current densities revealed a significant decrease in FE (<10% FE CO at 110 mA cm^–2^). The inability of catalysts produced in large batches to achieve high selectivity and durability at industrially relevant current densities is the key issue keeping these materials from commercialization. Low product yields and high energy usage in the synthesis process are secondary issues that also must be addressed.

In our previous work, we designed a simple method to synthesize Ni/Fe–N–C SACs.^33^ This catalyst was produced in a one-step process using two commercially available precursors—commercial multiwalled carbon nanotubes (MWCNTs) and melamine without the need for any pre- or post-treatment. Metallic (Ni and/or Fe) impurities present in commercial MWCNTs were leveraged as the metal source for the M–N–C catalyst; no addition of metal precursors is required. It is important to note that the synthesis of commercial MWCNTs is done at high temperatures, sometimes 1000 °C or higher, and although not included in the presented synthesis technique, this initial process should be considered when assessing material emissions and cost.^34^ In a typical procedure, as-received commercial MWCNTs and melamine (nitrogen precursor) are physically mixed, followed by a 2 h moderate-temperature (650 °C) pyrolysis in an inert atmosphere. Melamine as the N precursor has an advantage, as it has been shown that decreasing the N/C ratio of a nitrogen precursor can increase the N doping, with melamine resulting in ideal *N*-doping levels in carbon compared to other precursors like adenine and phenanthroline.^35^ Since no surface metallic nanoparticles are formed during the pyrolysis, the acid washing or post-treatment steps are circumvented completely. We accomplished 10 g batch synthesis with a 1:4 CNT to melamine mixing ratio, and the resultant catalyst achieved greater than 95% FE CO at 400 mA cm^–2^. It should be noted that this method does rely on premade CNTs, which are synthesized in a high-temperature process. This simple and scalable method motivated us to further close the gap for commercialization in three directions in this work: (1) further increasing the batch size, (2) reducing the melamine precursor, and (3) using an alternative, cheaper MWCNTs with more intrinsic metal impurities as the precursor, namely an industrial-grade MWCNTs that are typically available in the order of tonnes. In this work, we have achieved up to 75 g batch synthesis using both regular-grade MWCNTs (up to 5% impurity) and industrial-grade MWCNTs (up to 10% impurity) as the precursor and, meanwhile, significantly reduced the melamine consumption (CNT/melamine ratio reduced to 1:1). The catalytic performance of the large-batch catalysts was confirmed to be consistent with those from small batches, i.e., >98% Faradaic efficiency for CO production at industrially relevant current densities.

Another important aspect in evaluating the commercialization potential of eCO2R technologies is to rigorously quantify their economic and environmental impacts through techno-economic analysis (TEA) and life cycle assessment (LCA). Conventional process-based TEA and LCA typically emphasize the operation of eCO_2_R systems but often overlook the economic and environmental implications of catalyst synthesis.^5,36,37^ Moreover, many such studies extrapolate performance metrics obtained from catalysts synthesized at the milligram scale to deployment scenarios requiring gram-scale production or beyond.^37^ Others selectively adopt performance metrics from top-performing catalysts in the literature, without accounting for the diversity of active sites, dopants, and substrate materials that strongly influence both synthesis complexity and catalytic performance.^36,37^ This gap underscores the need for TEA and LCA frameworks that explicitly incorporate the catalyst type and synthesis pathway when evaluating the true viability of eCO_2_R technologies. In this work, we also performed TEA and LCA on our large-batch catalyst synthesis as well as the entire eCO_2_R process. Our results reveal clear economic and environmental benefits of our M–N–C catalyst and synthesis method over the status quo of benchmark Ag-based catalysts.

## 2 Experimental Methods

### 2.1 Materials

Standard-grade multiwalled CNTs (>95 wt % purity, Cheap Tubes Inc., referred to as Raw CNT) and industrial-grade multiwalled CNTs (>90 wt % purity, Cheap Tubes Inc., referred to as Raw indCNT) were used in this study, with Raw indCNT containing higher levels of residual metallic (Ni and Fe) impurities. Melamine (Mel) was acquired from Sigma-Aldrich and used as the nitrogen precursor in the catalyst synthesis process.

### 2.2 Synthesis of CNT-Mel and Derivatives under Different Batch Sizes

Four different catalyst samples have been prepared for this work: three using Raw CNT as the carbon precursor (denoted as CNT-Mel-*x*), where *x* represents the batch size in grams (i.e., CNT-Mel-25g, CNT-Mel-50g, and CNT-Mel-75g), and one using Raw IndCNT (denoted as indCNT-Mel-75g). Briefly, the synthesis of CNT-Mel-25g began with mixing 25 g of Raw CNT with an equal amount of Mel (i.e., a 1:1 mass ratio of CNT/Mel) using a standard kitchen-top blender. The resulting mixture was then packed into an 8 cm diameter quartz tube without a boat or crucible. Quartz wool and thermal blocks were placed in both ends of the tube to hold the mixture in place. The quartz tube was then placed in a tubular furnace (Thermal Scientific, Lindberg Blue M) and treated under an argon atmosphere (Airgas, UHP grade) at a constant flow rate of 50 mL min^–1^. The reactor temperature was ramped up at 5 °C min^–1^ until 650 °C and held at 650 °C for 3 h before naturally cooling down to room temperature. This exact synthesis procedure was employed for all four samples, regardless of the batch size or CNT source. The as-prepared catalysts were sampled at three different locations from the quartz tube, and their electrochemical performances were evaluated to assess the uniformness of catalyst quality.

### 2.3 Material Characterization

Sample morphology characterization was performed through scanning electron microscopy (SEM, JEOL JSM7500F) and transmission electron microscopy (TEM, FEI 200 kV Titan Themis STEM). High-angle annular dark-field scanning TEM/energy dispersive X-ray spectroscopy (HAADFSTEM/EDS) was performed on the same FEI 200 kV Titan Themis STEM. An EnviroESCA X-ray photoelectron spectroscopy (XPS) using an Al–Kα radiation source and an energy reference line from the C 1s (284.8 eV) of exogenous carbon was used for surface elemental quantification. Crystallinity approximations were measured from X-ray diffraction analysis (XRD, Bruker Lynxeye detector XTE). The Raman spectra of commercial and as-prepared powders were collected at room temperature using a Horiba XploRa Plus Raman microscope with a 532 nm diode-pumped laser, employing a 50× objective and a fixed laser power of 10 mW mm^–2^.

### 2.4 Electrochemical Performance Evaluation

#### 2.4.1 Flow Cell Testing

A standard three-compartment electrochemical flow cell was adapted for performing polarization sweeps and long-term testing on the prepared samples. The electrolyzer has two electrolyte pathways and one gas pathway. A 1 cm^2^ active area anode made of nickel foam, to facilitate the oxygen evolution reaction (OER), and a 1 cm^2^ active area carbon-paper-based cathode (Sigracet 39BB, Fuel Cell Store) are separated by an anionic exchange membrane (Fumasep PK 130, Fuel Cell Stores). The anolyte (20 mL/min) and catholyte (4.5 mL/min) were both 1.0 M KOH during polarization sweep testing and 0.5 M KHCO_3_ during long-term stability testing. The cathode was made through air spraying the catalyst ink atop the microporous layer of a gas diffusion layer (GDL, Sigracet 39BB). The ink was made of 3 mL of isopropyl alcohol, 300 μL of ionomer (PFSA Dispersion D5 5%, Fuel Cell stores), and 10 mg of catalyst. After 1 h of sonication, this ink was sprayed on a GDL to form an approximate 1 mg/cm^2^ catalyst loading based on the weight difference between prespraying and postdrying (60 °C for 2 h or room temperature overnight). CO_2_ (99.999%, Airgas) at 30 mL min^–1^ was flowed on the macroporous side of the GDL, permeating through the carbon paper and reacting at the triphase interface. Current to the cathode and anode was supplied by a DC power supply (Agilent 3633A), whereas a multimeter (Innova 3320) measured the potential between the working electrode (cathode) and reference electrode (Hg/HgO). None of the voltages presented had *iR* compensation.

Product gases from the electrolyzer were identified and quantified using an online gas chromatograph (GC-2010 Shimadzu), equipped with a thermal conductivity detector (TCD) for CO, H_2_, and CO_2_ measurement and a flame ionization detector (FID) for any hydrocarbon measurement. The Faradaic efficiency (selectivity) of each product is calculated from eq 1.

1 $$FE=\frac{zPFvC_{i}}{RTj}\times 100$$

where “*F*” represents the Faraday constant (∼96,485 C mol^–1^), “*v*” is the volumetric flow rate of CO_2_ supplied to the cell, “*j*” is the applied current (A), “*z*” is the number of electrons required to participate in a Faradaic reaction (i.e., *z* = 2 for CO or H_2_ production), “*P*” is the ambient pressure (101 kPa), “*R*” is the universal gas constant (8.314 J mol^–1^ K^–1^), “*T*” is the ambient temperature (298 K), and “*C*_*i*_” is the volumetric concentration of the target product as measured by the GC. For all samples electrochemically tested in this study, CO was the main product with minor H_2_ production, and there was no evidence of any hydrocarbon or oxygenate products through eCO_2_R.

#### 2.4.2 Electrochemical Surface Area Measurement

A conventional three-electrode H-Cell test setup was used to measure the electrochemical surface area (ECSA) of prepared electrocatalysts through the double-layer capacitance (C_DL_) method. Briefly, experiments herein entail a working (cathode), counter (anode for OER), and reference electrode (Ag/AgCl, 3 M KCl), all of which were submerged in the same chamber of 0.5 M KHCO_3_. The anode and cathode were not separated by an AEM, unlike conventional H-Cell tests, since the primary goal for this exercise was to attain ECSA of samples through cyclic voltammetry (CV) readings under a non-Faradaic potential window. Therefore, competing the hydrogen oxidation reaction (HOR) and the cathodically competing oxygen reduction reaction (ORR) were not considered. To prepare the working electrode for these tests, a catalyst ink constituted of 370 μL of isopropyl alcohol, 200 μL of deionized water, 30 μL of ionomer (PFSA Dispersion D5 5%, Fuel Cell stores), and 10 mg of catalyst. After 1 h of sonication, 200 μL of this catalyst ink was drop-cast onto a Toray 060 carbon paper to produce a 1 cm^2^ active area with 1 mg/cm^2^ of catalyst loading. The counter electrode was made up of a 1 cm^2^ Pt foil. Voltage (V) vs RHE measurements were reported using the Nernst relation (eq 2). Thirty sccm of CO_2_ (99.999%, Airgas) was bubbled into the catholyte for 30 min to ensure CO_2_ saturation of the electrolyte, and this flow rate was maintained throughout testing. Finally, a Biologic potentiostat was used to power the cell and gather electrochemical readings.

2 $$E_{RHE}=E_{ref}+0.059\times pH+E_{ref}^{0}$$

#### 2.4.3 Membrane Electrode Assembly Testing

An MEA (or zero-gap electrolyzer) with a 5 cm^2^ effective area was used for stability testing on the CNT-Mel-75g sample. The cathode was prepared through the same method used for flow cell testing as previously described, except with a larger area. The anode was a 5 cm^2^ Ni foam. A Sustainion X37-50 grade *RT* membrane was used. No catholyte was used while 200 mL min^–1^ of CO_2_ was bubbled through deionized water before it flowed through the cathodic flow field. The anolyte used was 1 M KOH flowing through the anodic flow field at 15 mL min^–1^. A Keysight E36313A power supply was used to apply the current to the electrolyzer. Gaseous products from the cathodic flow field were measured by GC.

### 2.5 Commercialization Potential Analyses

#### 2.5.1 Techno-Economic Analyses

Following Badreldin et al.,^36^ we simulate the cash flow and net present value of a representative plant with a 20 year life span, producing 100,000 kg of CO per day from CO_2_ electrolysis, operating 350 days per year with a return on investment (ROI) of 10%. The minimum selling price (MSP) was calculated with capital, labor, land, and other operational costs. The only cathodic byproduct considered in the model is H_2_, while O_2_ is considered as the anodic byproduct. MSPs are calculated under two scenarios with and without consideration of the sale of these byproducts. The operational and capital costs of the separation of the cathodic H_2_ are considered regardless of the scenario with unreacted CO_2_ in the cathodic exit stream recycled. This unreacted CO_2_ must therefore be separated from both byproduct H_2_ and the desired CO gas. For separation, pressure-swing absorbers (PSAs) are used. The technology modeled in this work is a sequential CO_2_ amine-based capture, stripping, compression, and electrochemical reduction integrated system. A 38.9% income tax and a 10 year modified accelerated cost recovery system (MACRS10) are used for property depreciation. The system boundary for the TEA is shown in Figure S1, comparative electrochemical factors are shown in Table S1, and system and financial assumptions are given in Tables S2 and S3, respectively.

#### 2.5.2 Life Cycle Assessment

A cradle-to-gate LCA was performed in accordance with the ISO 14040/14044 standards^38,39^ to quantify the greenhouse gas emissions associated with CO_2_ electroreduction to CO. The study followed the standard framework of goal and scope definition, life cycle inventory (LCI), and impact assessment, with greenhouse gas emissions expressed as grams of CO_2_-equivalents (g CO_2_e) as the primary indicator. The total emissions are estimated by

3 $$CO_{2eTotal}=\sum \left(E_{i}\times EF_{i}\right)$$

where *E*_*i*_ is the activity data for process *i* (e.g., electricity consumption, material input, or direct emission), and *EF*_*i*_ is the corresponding emission factor for process *i*. Primary process data (Table 1) was obtained from experimental results and process modeling and supplemented with secondary data from established databases^40^ and relevant literature to address data gaps. Material emissions factors used in calculations are shown in Table S5. The system boundaries (Figure S1) encompassed all major capture and conversion process stages, from raw material inputs and energy use to conversion and separation processes. In addition, scenario analysis was carried out to evaluate the influence of catalyst batch size and material choice on overall emissions, enabling comparison across alternative process configurations.

**Table 1:** CO_2_e Emissions for the Operation and Synthesis of CNT-Mel and Ag Nanoparticles at the Base Case 50% SPCE Using Standard Grid Electricity

	CNT-Mel gCO_2_e/gCO	Ag NPs gCO_2_e/gCO
utilized CO_2_	–1.57	–1.57
catalyst synthesis	4.59 × 10^–5^	1.36 × 10^–4^
CO_2_ electrolysis	2.37	3.10
separation	0.399	0.399
CO_2_ capture	0.720	0.720
CO_2_ compression	0.954	0.954
net emissions	2.88	3.61

## 3 Results and Discussion

### 3.1 Materials Characterization

Various material characterization techniques were conducted to confirm the uniformity of different batch sizes of this Ni/Fe–N–C catalyst in terms of both morphology and elemental composition. A major difference between this work and our previous study^33^ is the scale up of the batch synthesis and the use of less amounts of precursor materials. Previously, a 1:10 mass ratio of CNT/melamine was used for mg-scale batches, and a 1:4 ratio was used for the 10 g batch. The current work achieves a similar catalytic structure with a mere 1:1 mass ratio of CNTs to melamine at a batch size of up to 75 g. Morphological analyses were performed through transmission electron microscopy (TEM) for CNT-Mel-75g (Figure 1a), indCNT-Mel-75g (Figure S2a), Raw-CNT (Figure S3a), and Raw-indCNT (Figure S4a). In all cases, the characteristic tubular morphology of CNTs was clearly observed, confirming that the 1-D CNT structure remained intact after pyrolysis. Furthermore, no significant structural differences were noted between regular-grade CNTs (>95% carbon, Raw-CNT) and industrial-grade CNTs (>90% carbon, Raw-indCNT), underscoring the robustness of the synthesis method across different precursor grades. Importantly, no residual melamine was detected for either CNT-Mel-75g or indCNT-Mel-75g synthesis, indicating complete thermal decomposition of the nitrogen precursor. Figure 1b depicts a high-resolution TEM image of CNT-Mel-75g that includes circled bright spots. These spots indicate the presence of single-atom metal–nitrogen sites, either NiN_4_ or FeN_4_. Figure S2b shows the high-resolution TEM image of indCNT-Mel-75g, which displays similar bright spots indicating that single-atom sites are present in this sample as well. These single-atom sites are unique to the melamine-pyrolyzed CNTs, as the Raw CNT and Raw indCNT TEM images (Figures S3b and S4b) are absent of such bright spots. The complete thermal decomposition of melamine is further supported by the elemental mapping of N for CNT-Mel-75g (Figure 1c) and indCNT-Mel-75g (Figure S2c), as the N mapping clearly follows the same tubular structure as the CNT, strongly suggesting a uniform and homogeneous distribution of N doping throughout the surface of CNTs. Similarly, Ni and Fe mappings are uniform throughout the surface of the treated CNTs without obvious zones of localization or phase separation (i.e., nanoparticle formation). The above material characterization confirms the formation of Ni/Fe–N–C catalysts from the large-batch synthesis using both regular-grade or industrial-grade Raw CNTs as the precursor.

**Figure 1:**
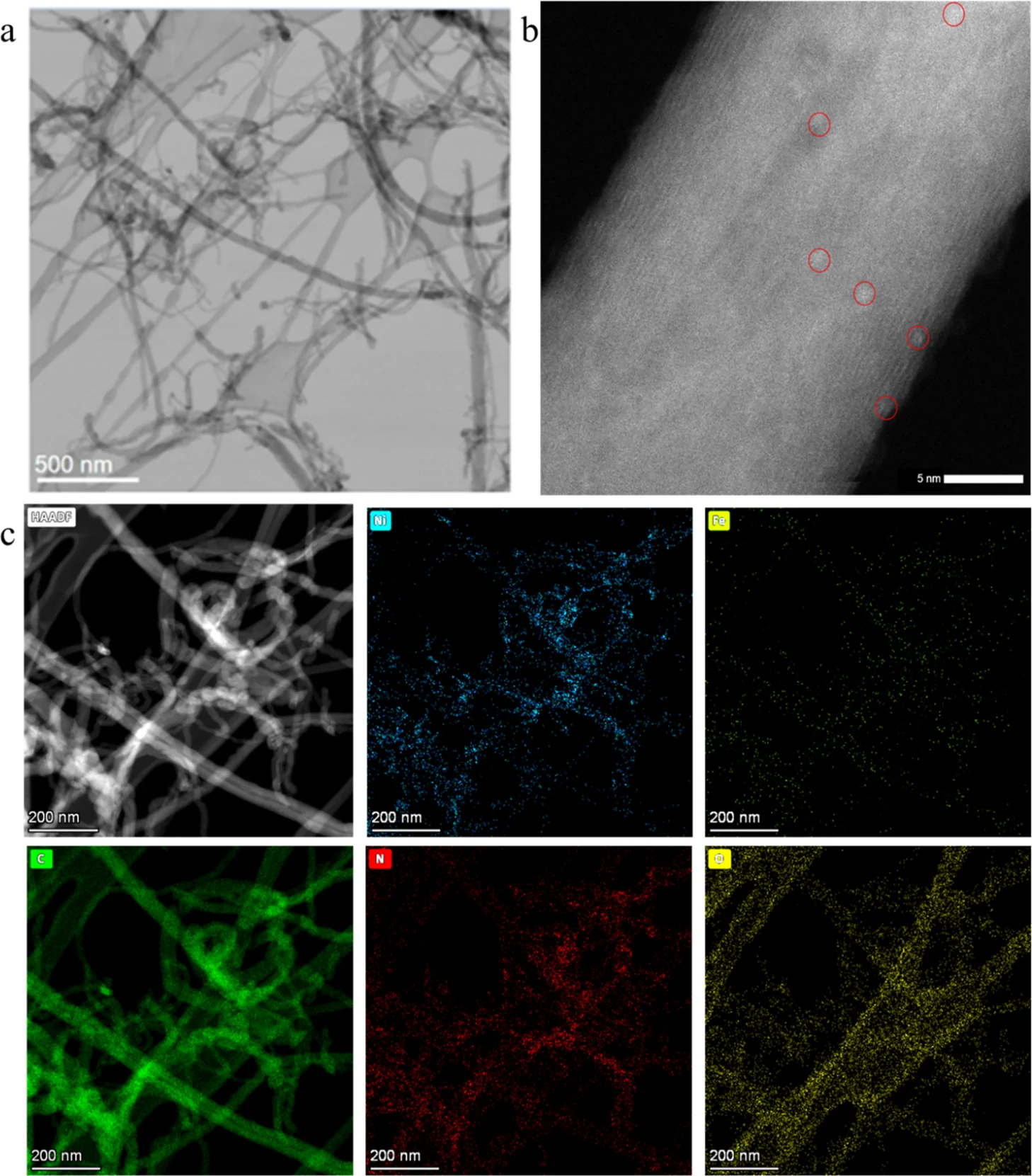
(a) Bright-field TEM, (b) high-resolution TEM, and (c) HAADF-STEM and corresponding elemental maps of CNT-Mel-75g.

While electron microscopy revealed the preservation of CNT morphology and atomic dispersion of active sites, complementary X-ray photoelectron spectroscopy (XPS) and X-ray diffraction (XRD) analyses were employed to probe the surface chemistry, bonding environments, and bulk crystallinity of the materials. XPS was performed to probe the N loading, coordination, and M–N–C character differences between samples of different batch sizes. Figure 2a shows a Ni 2p peak at 856 eV, a Fe 2p peak at 715 eV, a O 1s peak at 534 eV, and a N 1s peak at 399.5 eV, all of which are within range of the respective literature.^41–44^ As shown in Table S4, N concentration was in the range of 2.0–2.9 at. % for the samples, with a slight increase in N content with batch size. In contrast, only 1.3 at. % of N concentration was reported in our previous work, which employed a 1:10 CNT/melamine mass ratio in the synthesis of a 100 mg batch.^33^ Despite using a much lower CNT/melamine mass ratio (1:1), the higher N-concentration obtained in this work can be explained by a much longer residence time of melamine decomposition products (mainly ammonia gas) in the reactor that are responsible for N-doping through ammonia-CNT interaction during pyrolysis. The longer residence time is attributed to (1) a lower Ar flow rate at 50 mL cm^–2^ compared to 80 mL cm^–2^ in the previous work, and (2) a much larger batch (up to 75 g) compared to 100 mg in the previous work, which significantly increased the packing volume of the raw materials. The N 1s spectra for CNT-Mel-75g are shown in Figure 2b, and the deconvolution fitting indicates that most of the doped N is coordinated as M–N bonds (M = Ni or Fe) at a 399.8 eV, further supporting the conclusion of M–N–C site formation.^45^ The N 1s spectra for indCNT-Mel-75g shown in Figure 2c show a similar dominance of M-N bonds, proving the use of industrial CNTs does not have a major impact on the coordination of N in the carbon nanotube structure. The major difference between the N 1s spectra of CNT-Mel-75g and indCNT-Mel-75g is the prominence of pyridinic N. There is much more pyridinic N present in indCNT-Mel-75g, whereas CNT-Mel-75g has very little pyridinic N; instead, larger coordination’s of graphitic and pyrrolic N are present. This is most likely due to the less coordinated, high-impurity Raw indCNTs used to produce indCNT-Mel-75g, which provide less coordinated carbon for graphitic and pyrrolic coordination to occur.^46^

**Figure 2:**
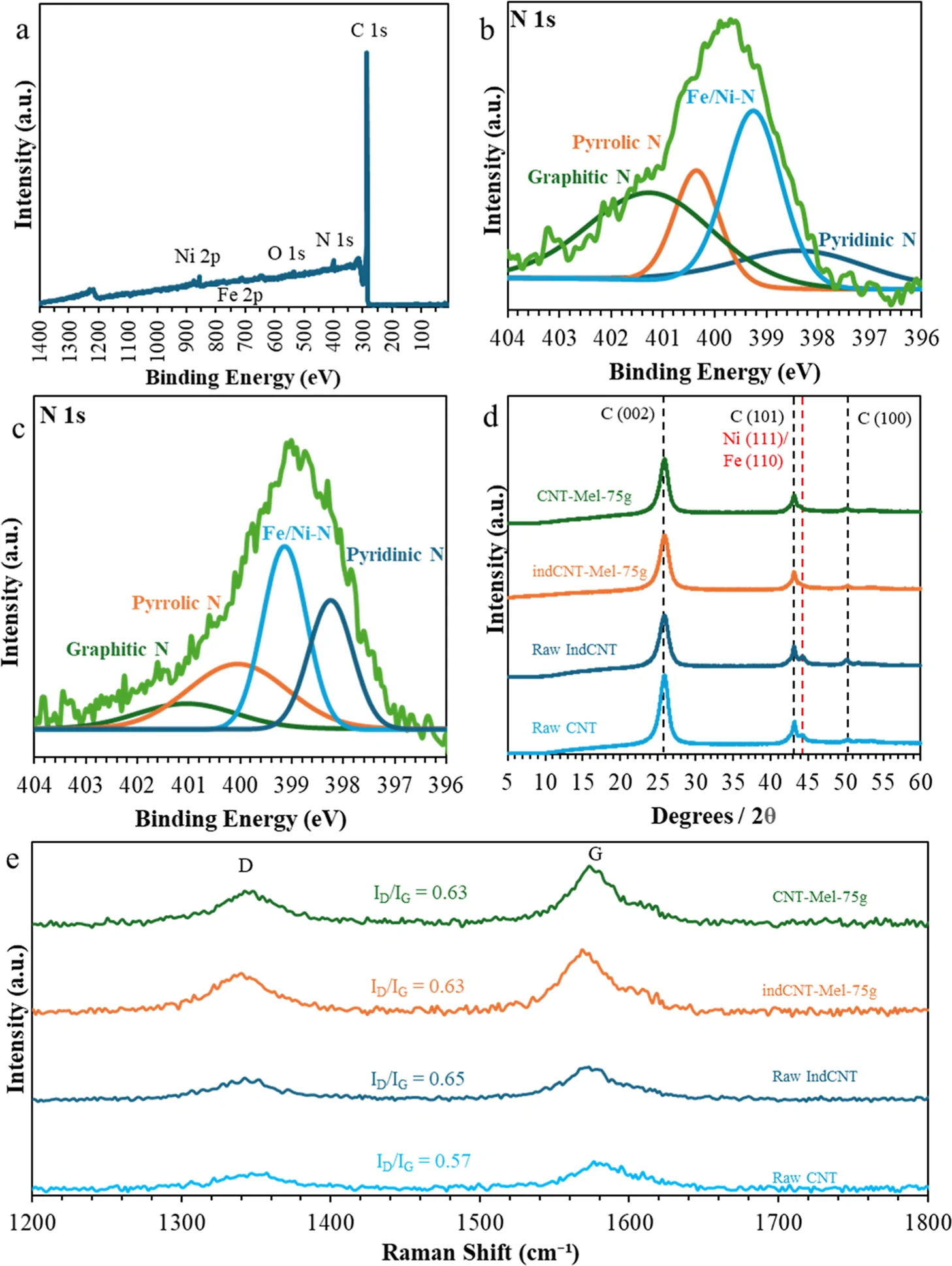
(a) Full survey XPS spectra for CNT-Mel-75g; (b) N 1s spectra for CNT-Mel-75g; (c) N 1s spectra for indCNT-Mel-75g; (d) XRD patterns for Raw-IndCNT, RawCNT, IndCNT-Mel-75g, and CNT-Mel-75g; and (e) Raman spectra for Raw-IndCNT, RawCNT, IndCNT-Mel-75g, and CNT-Mel-75g.

X-ray diffraction (XRD) was also performed to characterize the crystal structure of the large batch samples in comparison to the Raw CNTs (Figure 2d). All four samples show a major peak at a 2θ of ∼26°, corresponding to the (002) diffraction peak of carbon. The major difference in the XRD spectra between samples exists between the raw and treated batches. Briefly, a small peak is present in the Raw-CNT and Raw-indCNT diffractograms at 2θ of approximately 44° which corresponds to an overlapping combination of metallic Ni(111) and Fe(110) facets.^47^ Their decreased prominence in the treated CNT-Mel-75g and indCNT-Mel-75g samples suggests the redistribution of the metals on the surface as M–N–C characters. Since the M–N–C character does not have a unit cell or repeatable lattice, its identification through XRD is not feasible.^47^ This can also explain the XPS results (Table S4) that present an increase in surface metal content after pyrolysis. It is noted that the peak at 44° is more prominent in the Raw-indCNT sample, likely due to its higher content of metal impurities than in Raw-CNT. Because of the overlap between Ni(111) and Fe(110) reflections in XRD and the limited resolution of Ni–N and Fe–N species within the N 1s XPS region, unambiguous differentiation between Ni–N_*x*_ and Fe–N_*x*_ coordination environments is not feasible in this study. Nevertheless, based on the higher surface atomic fraction of Ni relative to Fe determined by XPS, combined with the expectation that both metals preferentially adopt thermodynamically stable M–N_4_ configurations within N-doped carbon matrices,^48^ a reasonable assessment of their relative contributions can be made. Prior experimental and computational (density functional theory) studies have shown that Ni–N_4_ sites exhibit higher intrinsic selectivity and activity for CO_2_-to-CO conversion compared to Fe–N_4_ analogues.^49^ Taken together, the greater surface prevalence of Ni and its more favorable catalytic characteristics strongly suggest that Ni–N_*x*_ moieties are the dominant active sites responsible for the observed electrochemical performance. Fe–N_*x*_ sites may contribute to a secondary capacity in this work, or they can play a dominating role if the Raw CNTs have a prevalent Fe impurity over Ni.

Raman spectroscopy was also performed to evaluate the structural integrity and defect characteristics of Raw CNT, Raw indCNT, and their corresponding treated samples. The carbon framework was assessed using the I_D_/I_G_ ratio, defined as the intensity ratio of the D band (∼1350 cm^–1^), associated with defects and disordered carbon, to the G band (∼1580 cm^–1^), corresponding to graphitic sp^2^ carbon.^50,51^ As shown in Figure 2e, all samples exhibit similar *I*_D_/*I*_G_ ratios of approximately 0.6, indicating that the overall balance between defect and graphitic domains is largely preserved following treatment. The slightly higher I_D_/I_G_ ratio observed for Raw indCNT relative to Raw CNT is consistent with its lower initial purity and higher defect density. Notably, both CNTs converge to similar *I*_D_/*I*_G_ values after treatment, suggesting that the pyrolysis and melamine-assisted nitrogen incorporation establish a common structural equilibrium that overrides initial differences in defect density. An increase in the absolute intensity of both D and G bands is observed for treated samples, which is attributed to the combined effects of nitrogen incorporation, formation of surface metal coordination sites, and enhanced graphitization during pyrolysis. Overall, these results indicate that the synthesis process preserves the graphitic framework while introducing controlled defect sites associated with M–N–C formation.

### 3.2 H-Cell Testing Results

H-cell testing was performed to measure the electrochemical activity of catalysts prepared at different batch sizes, with the aim of assessing whether samples synthesized through the same protocol exhibit consistent performance metrics irrespective of batch size. Furthermore, any difference in activity between CNT-Mel and indCNT-Mel samples would capture the influence of the marginally higher metallic content observed in Raw-indCNTs compared to the Raw-CNT counterpart. Moreover, to evaluate whether active site density varied with batch size, the electrochemical surface area (ECSA) of the samples was determined by plotting the scan rate against the corresponding change in current density between forward and reverse sweeps of a CV plot in a non-Faradaic potential window (0.15–0.25 V vs RHE) over scan rates ranging from 20 to 120 mV s^–1^ (Figure S5a–d), as shown in Figure 3a. The slope of the plot shown in Figure 3a corresponds to the double layer capacitance (*C*_DL_). Consequently, ECSA is obtained as the ratio between *C*_DL_ and specific capacitance (*C*_S_, taken as 0.04 mF cm^–2^ for 0.5 M KHCO_3_).^52^ It is found that *C*_DL_ and ECSA increase with batch size. While the difference between the 25 and 50 g batches was negligible, the 75 g batches exhibited a 14% higher ECSA compared to the 50 g batch of CNT-Mel. Moreover, the indCNT-Mel-75g batch exhibits the highest *C*_DL_ and ECSA, consistent with its elevated metal content as revealed by XPS (Table S4). LSV curves taken in Figure 3b show that CNT-Mel-25g is marginally more active than the other three samples, with indCNT-Mel-75g exhibiting the second–best activity. However, and of key importance, the non-*iR*-compensated onset overpotential (η_0_) (taken at a low current density of 5 mA cm^–2^) for all samples is ∼0.42 V (vs RHE). This suggests that all samples have practically the same electrochemical intrinsic activity, as η_0_ is the most reliable performance metric in H-cell measurements, where transport limitations dominate at higher current densities. Further, upon normalizing the polarization curves in Figure 3b with the ECSA values obtained in Figure 3a, the ECSA-normalized LSV plots in Figure 3c reveal that at higher current densities, individual active sites are less active for indCNT-Mel-75g and CNT-Mel-75g and more active for CNT-Mel-50g and CNT-Mel-25g. However, onset overpotentials in the ECSA-normalized polarization plots Figure 3c are all consistent. This discrepancy suggests that the CNT-Mel-25g and CNT-Mel-50g samples contain active sites that are less transport-limited than their counterparts, effectively suggesting a degree of lower coordination Ni/FeN_4‑*x*_ in the 75 g batch. Notwithstanding, the comparable ECSA values and consistent onset electrochemical activities attained for the examined samples herein suggest that scaling the synthesis to 75 g is effective.

**Figure 3:**
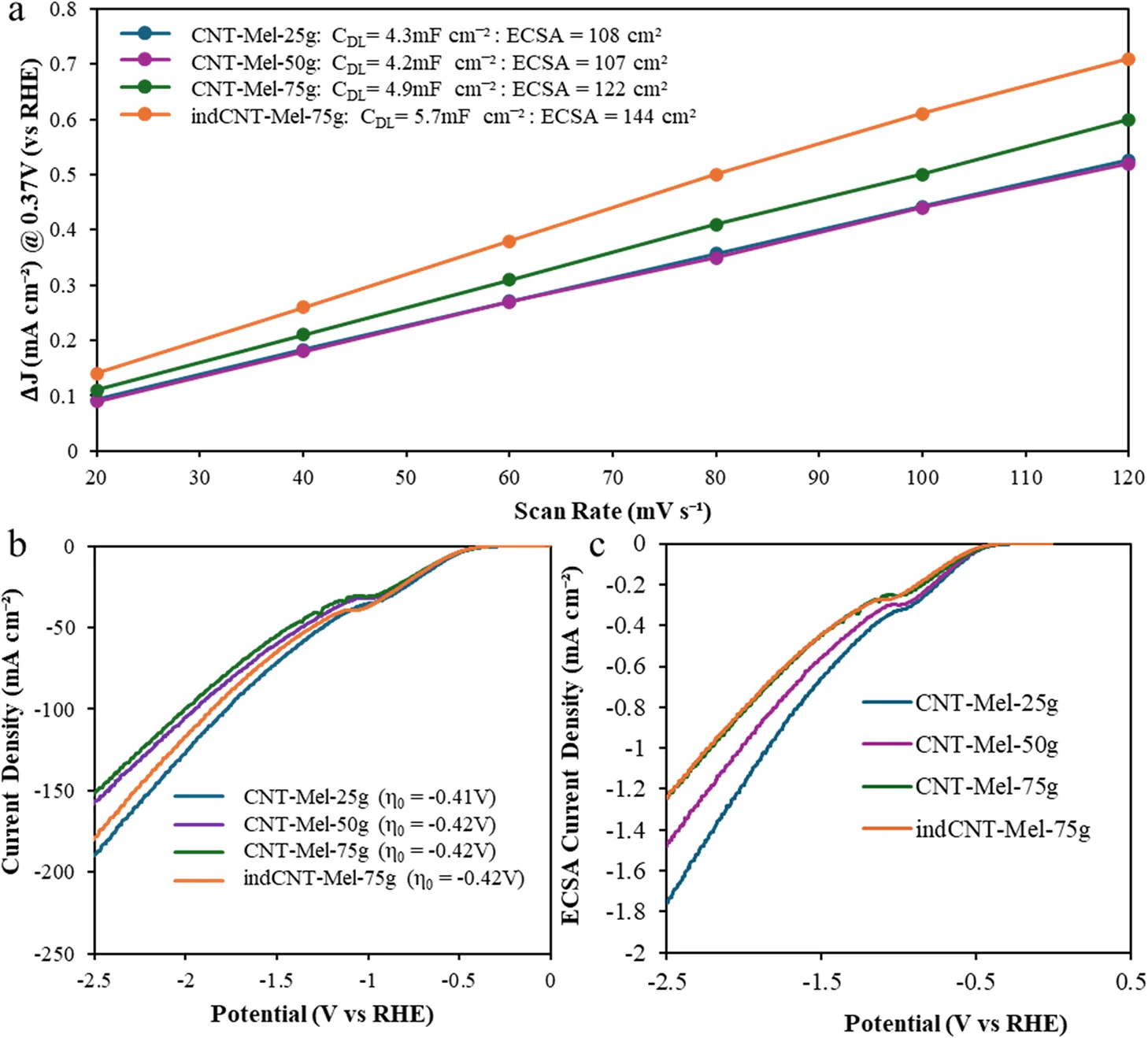
(a) Scan rate versus changes in current density in a non-Faradaic potential range to determine C_DL_ and ECSA; (b) non-*iR*-corrected LSV under 0.5 M KHCO_3_; and (c) ECSA-normalized LSV for different batches of CNT-Mel and indCNT-Mel-75g.

### 3.3 Flow Cell Testing Results

In eCO_2_R chemistry, FE toward the target product is a key performance metric, as it directly affects the downstream separation costs of electroreduction products and therefore the overall process economics.^53^ Since active site configuration strongly influences selectivity, it is essential to evaluate FE under industrially relevant current densities for batches of different sizes. To that end, flow cell testing was employed to characterize FE and validate the scalability and applicability of the CNT-Mel catalysts beyond H-cell testing. Figure 4 presents the current density sweep results of CNT-Mel-25g, CNT-Mel-50g, CNT-Mel-75g, and indCNT-Mel-75g. Across all samples, the FE toward CO production (FE_CO_) remained above 98% from 100 to 500 mA cm^–2^, with the corresponding applied potential increasing linearly with current density and exhibiting minimal variance among the samples. Error bars on the FE and potential values represent three electrodes made of three samples of catalyst collected from different locations of the pyrolysis chamber, i.e., near the front, middle, and end of the chamber. Consistent data and small error bars from three different catalyst samples affirm that the location in the pyrolysis chamber has no impact on results and the property of the catalyst in the large batches is uniform throughout the chamber. It is also noted that the non-*iR* corrected potential was as low as around −2.0 V vs RHE at 500 mA cm^–2^, significantly outperforming other large batch synthesized catalysts reported in the literature, not to mention that most of those reports performed 80–95% *iR* corrections.^22,24–27^ The peak partial current density of CO (*j*_CO_) achieved in this work was at ∼500 mA cm^–2^, with all samples peaking above 480 mA cm^–2^. This performance markedly surpasses those of large-batch M–N–C catalysts reported in the literature, with the highest value of *j*_CO_ at 360 mA cm^–2^ and the rest below 100 mA cm^–2^.^22,24–27^

**Figure 4:**
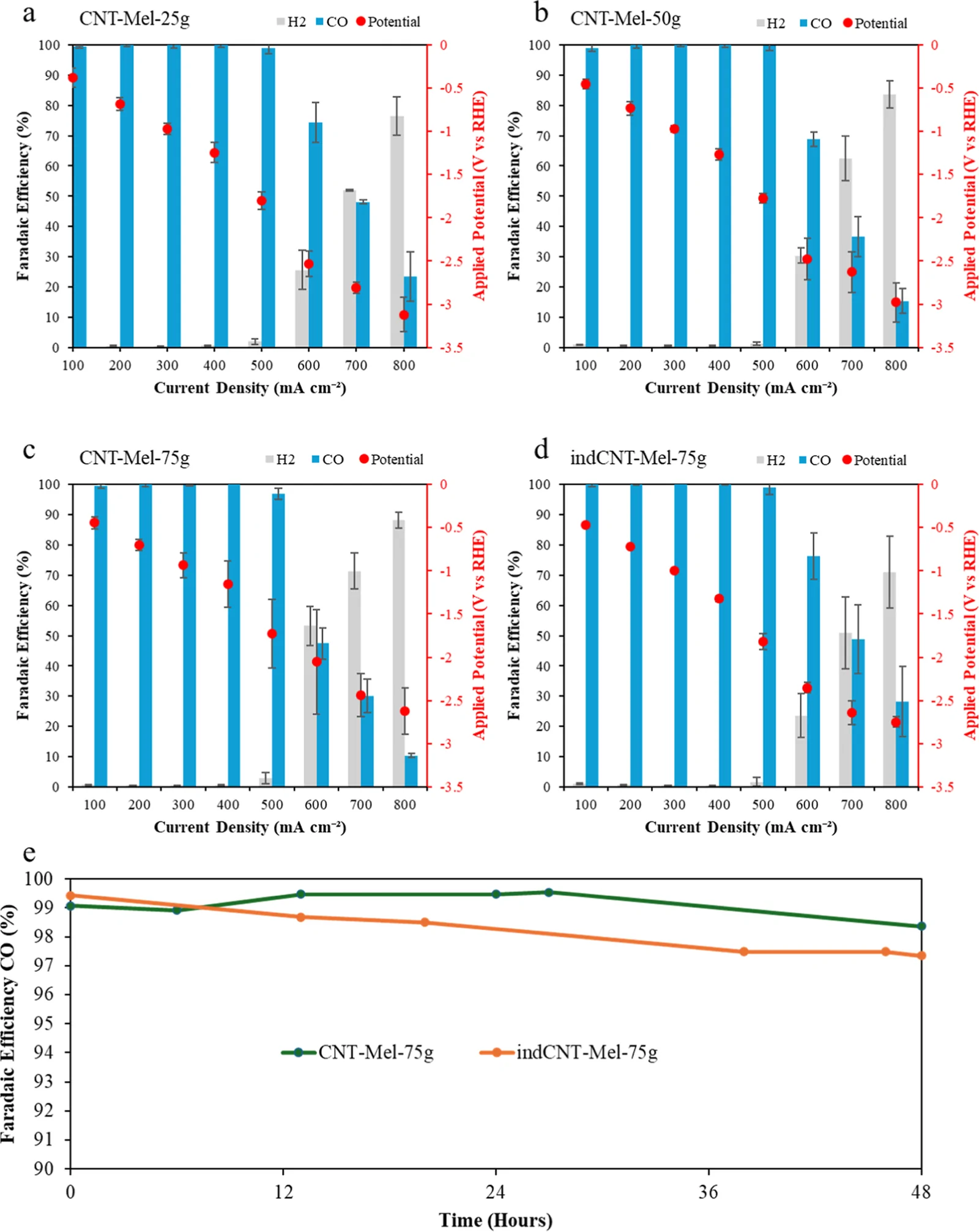
Faradaic efficiency and applied potentials for CO_2_ to CO conversion by (a) CNT-Mel-25g, (b) CNT-Mel-50g, (c) CNT-Mel-75g, and (d) indCNT-Mel-75g in a flow cell; (e) 48 h stability test in a flow cell at a constant current density of 100 mA cm^–2^ using CNT-Mel-75g and indCNT-Mel-75g samples.

The eCO_2_R performance of our samples began to vary only at 600 mA cm^–2^ and above. At these higher current densities, the competing HER became more prominent due to the limitation of CO_2_ transport toward the eCO_2_R. HER has a compounding negative impact on the performance of these samples, resulting in the presented low FE_CO_ and larger error bars at high current densities (>500 mA cm^–2^).^54^ No significant differences between batch sizes can be discerned from these results, indicating that the synthesis method is reliable for different batch sizes. The larger error bars at the high current density range (greater than 500 mA cm^–2^) should not be ascribed to substantial variation in catalyst properties because they would have manifested in the 100–500 mA cm^–2^ range. The overall performance of the indCNT-Mel-75g batch was similar to those of the other three batches made from regular-grade CNTs, suggesting the synthesis method is effective for all types of Raw CNTs that have metal impurities. This is a significant finding because industrial-grade CNTs are much cheaper than regular-grade CNTs, making them a more cost-effective option for large-scale synthesis and eCO_2_R applications.

Performance stability is another important metric to assess the potential for commercialization. Figure 4e presents the 48 h stability test results on both CNT-Mel-75g and indCNT-Mel-75g. Both samples maintained >97% FE_CO_ throughout the 48 h test with no discernible difference in performance, indicating good stability of both catalysts. Other noncatalyst factors such as GDL design, electrolyzer type, CO_2_ flow rate, and membrane and electrolyte choices are believed to be equally important as the catalyst design to sustain hundreds of hours of stability.^55,56^ This work focuses on scalable synthesis and validation of catalyst uniformity and effectiveness; future work will focus on the optimization of the various system parameters to achieve longer-term stability at higher current densities.

## 4 Commercialization Potential Evaluation

### 4.1 Techno-Economic Analyses

The TEA was conducted to benchmark the full-cell performance of the large-batch CNT-Mel catalyst against status-quo Ag nanoparticle (NP) eCO_2_R cathodes (see Supporting Information Note 1: CNT-Mel-75g TEA Example Calculation). Its techno-economic metrics are directly compared with a high-performing Ag NP system recently reported by Ko et al.^57^ An Ag-based catalyst was selected as the benchmark cathode owing to its well-established large-scale manufacturability and proven industrial relevance, underpinned by thousands of hours of demonstrated operational stability in CO_2_ electrolyzers.^58^ A net-present-value (NPV)-based TEA model is used to emulate a conventional sequential CO_2_ capture, stripping, compression, and electrochemical reduction route.^36^ Valuable H_2_ (1900 USD/tonne) gas is produced as a byproduct of eCO_2_R to CO, although with a much lower selectivity than CO, whereas O_2_ (400 USD/tonne) is produced anodically. The sale of H_2_ and O_2_ byproducts could have a large impact on the economic viability of the eCO_2_R systems.^36,59^ However, in actual eCO_2_R commercial deployments, the sale of these byproducts may be unreasonable due to a lack of demand and high transport costs depending on the implementation. Therefore, MSP analyses considered in the NPV model were done with the sale of byproducts (Figure 5a) and without the sale of byproducts (Figure 5b).

**Figure 5:**
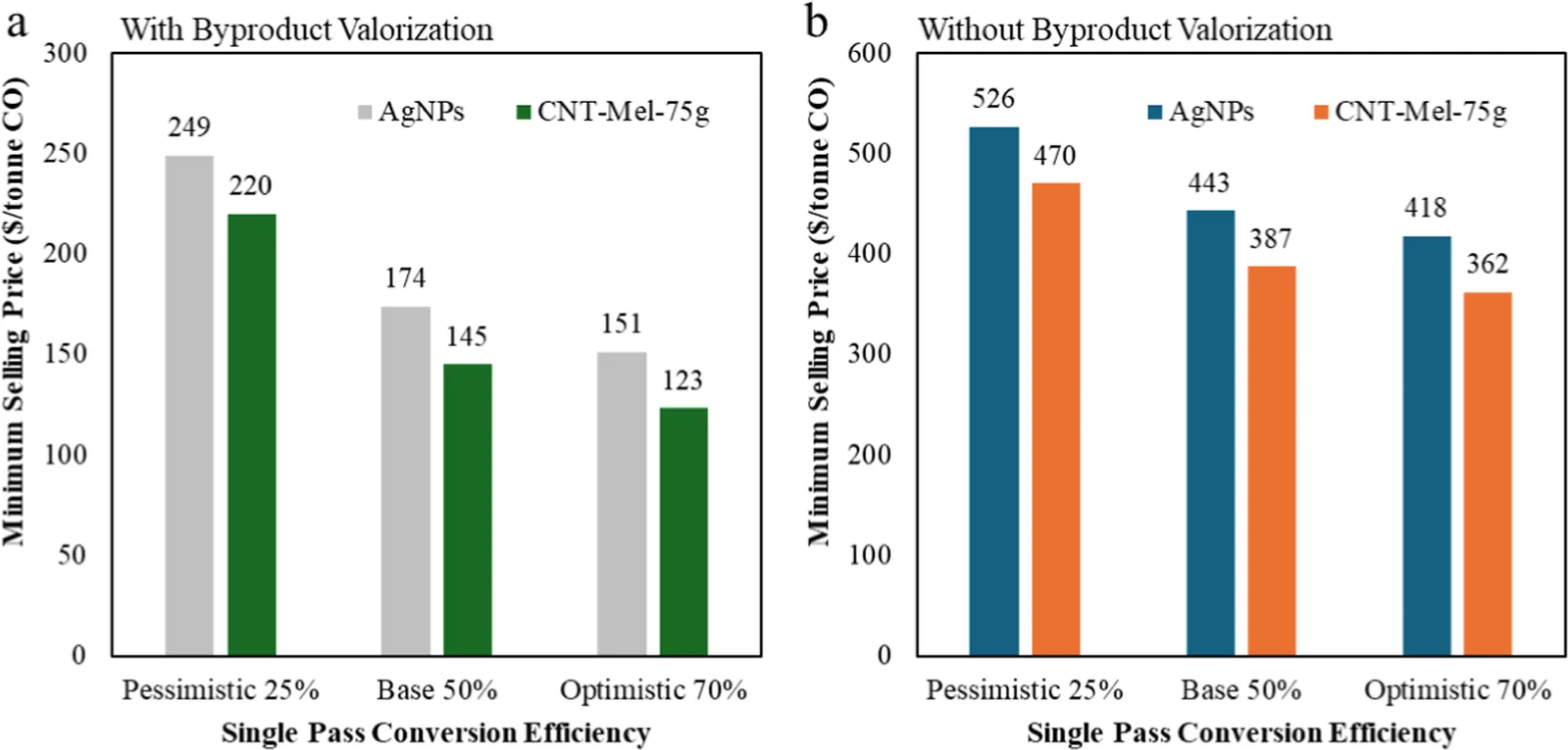
(a) Minimum selling price of 1 tonne of CO with sale of byproducts considered and (b) minimum selling price of 1 tonne of CO without considering sale of byproducts using AgNPs^57^ and CNT-Mel-75g, respectively, as the catalysts.

There are five main electrochemical performance metrics that impact the operational cost of an integrated carbon capture and conversion system. Namely, they are (1) the operational single-pass conversion efficiency (SPCE), (2) the carbonate formation rate, (3) the operational current density, (4) the corresponding applied full-cell voltage, and (5) the attained product selectivity. These metrics for both large-batch CNT-Mel (i.e., CNT-Mel-75g) and Ag NPs are listed in Table S1 for comparison and are used in the associated TEA calculations. Conventional benchmark Ag-based NPs by Ko et al. achieved 91% FE toward CO at 3.4 V under 300 mA cm^–2^ of current density.^57^ To compare as directly as possible between in-house CNT-Mel-75g and contemporary literature benchmark Ag-based GDEs, a zero-gap electrolyzer test was conducted on CNT-Mel-75g using the same operational 300 mA cm^–2^ current density noted for Ag-based GDEs. We obtained a selectivity of 98% toward CO, which required an average of 2.8 V full-cell voltage (Figure S6). Moreover, a crucial performance metric for eCO_2_R is the SPCE, which depends not only on catalyst selectivity but also on system-level parameters, including the CO_2_ feed rate, electrolyzer active area, and operating current density. For TEA in this study, sensitivity analyses are conducted using base (50%), optimistic (70%), and pessimistic (25%) SPCE values reported by Badreldin et al.^36^ A carbonate formation rate of 50% is assumed and used for both CNT-Mel and Ag NPs, reflecting their shared electrolyte and CO target production rates.^60^ All other catalyst-independent processes and economic assumptions are summarized in Tables S2 and S3, respectively. The significant CO_2_ loss arising from carbonate formation is primarily a consequence of the operation in alkaline electrolytes. In contrast, acidic CO_2_ electrolysis systems have been shown to achieve a carbon utilization or conversion efficiency exceeding 80%.^61,62^ This enhanced CO_2_ utilization reduces the burden on downstream separation and recycling units, thereby lowering the energy demand and operational costs associated with the PSA and CO_2_ compressors. In the present study, alkaline conditions were employed due to catalyst-specific considerations, namely, the CNT-Mel-75g system comprising of atomically dispersed metal sites on a carbon-based support, which are susceptible to demetalation and carbon corrosion under acidic environments.^63,64^

Under the base 50% SPCE scenario with byproduct valorization, CNT-Mel-75g achieves a CO MSP of 145 $/ton, compared with 174 $/ton for Ag-based cathodes. CNT-Mel-75g outperforms Ag NPs across all scenarios, driven by its lower operating full-cell voltage and higher selectivity measured in zero-gap electrolyzer tests. When byproducts are valorized, both catalyst systems achieve MSPs below the CO market price (400$/ton^59^) in all three SPCE cases. However, without byproduct sales, only CNT-Mel-75g remains economically feasible, and only when operating at the base or optimistic SPCE scenario. The economics of Ag NPs are intrinsically constrained by their lower CO selectivity. Byproduct valorization, therefore, plays a central role in determining the economic competitiveness of eCO_2_R system deployments, independent of the catalyst choice. Further research is needed to assess how byproduct sales to established or niche markets will shape the geographic and industrial deployment landscape of the eCO_2_R technologies.

### 4.2 Life Cycle Assessment

The LCA shows that the CNT-Mel delivers environmental advantages over the benchmark Ag NP catalyst for commercial eCO_2_R. The LCA (Supporting Information Note 2: CNT-Mel-75g LCA Example Calculation) accounts for five primary emission sources, namely, catalyst synthesis, electrolysis, product separation, compression, and CO_2_ capture. Table 1 reports the resulting carbon emission intensity (*g*_CO_2e__/*g*_CO_) together with a breakdown of contributions across the system boundary. Grid electricity used for Table 1 was modeled using the Ecoinvent 3.10 database, comprising approximately 49% natural gas, 24% renewables, 10% nuclear, 17% lignite, and less than 1% other sources.^65^ The shares are based on 2022 statistics for the US-TRE region from the U.S. EPA Emissions and Generation Resource Integrated Database (eGRID). Grid losses and imports were also taken from the 2022 eGRID data set. This framework enables a fair and policy-relevant comparison of catalyst technologies under conditions representative of near-term industrial deployment.

Since unreacted CO_2_ is recycled in the modeled system, CO_2_ utilization remains constant across catalysts, and differences in conversion efficiency are reflected instead in the electrolysis energy demand. Catalyst synthesis emissions for Ag NPs are relatively high, driven by emission-heavy silver mining and refining, along with energy-intensive nanoparticle synthesis routes such as flame spray pyrolysis.^66^ In contrast, CNT-Mel exhibits lower synthesis emissions owing to its much lower metal loading and the use of earth-abundant and accessible Fe and Ni (Table S5 for catalyst input emission factors).^67^ Moreover, scaling CNT synthesis significantly reduces emissions per unit of catalyst, underscoring the importance of sustainable, scalable manufacturing routes for industrial implementation. Nevertheless, the catalyst synthesis contributes only a minor fraction of total life cycle emissions, which are dominated by electrolysis, CO_2_ capture, and product separation. These results indicate that catalyst development at scale should prioritize high selectivity and low overpotentials, as these parameters ultimately govern system-level CO_2_e emissions.

As shown in Table 1, electricity consumption during CO_2_ electrolysis constitutes the dominant source of life cycle emissions for both catalysts. Electricity consumption in eCO_2_R is determined by five main performance metrics, which include product selectivity, full-cell voltage, current density, carbonate formation rate, and SPCE. CNT-Mel outperforms Ag NPs in both CO selectivity and full-cell voltage, whereas SPCE, current density, and carbonate formation rate were held constant for both catalysts. A detailed breakdown of the cell performance metrics for both systems is provided in Table S1. The lower electrolysis energy demand of CNT-Mel accounts for nearly all of the emission difference between the two catalysts, highlighting the central role of cell-level performance in determining the environmental footprint of large-scale eCO_2_R systems. Energy requirements for separation are nearly constant across materials, as PSA operation energy requirements depend on volumetric flow of output gases, which is mostly dependent on SPCE and carbonate formation, both of which have been held constant. CO_2_ capture and compression processes occur before the CO_2_ enters the cell; therefore, their emissions are also catalyst-independent.

When using standard grid electricity, the net CO_2_e emissions are lowest when using CNT-Mel, which emits roughly 2.88 g_CO2e_/g_CO_. The use of AgNPs leads to higher emissions of 3.61 g_CO2e_/g_CO_, a 25% increase in emissions compared to CNT-Mel. Carbon monoxide is conventionally produced via steam methane reforming (SMR), a high-temperature, natural-gas-driven process.^68^ Despite its fossil origin, SMR exhibits a relatively low carbon intensity of 1.40–2.32 gCO_2_e per gCO,^68^ indicating that the carbon footprint of CO production is strongly governed by the electricity mix used in alternative eCO_2_R pathways, as shown in Figure 6.

**Figure 6:**
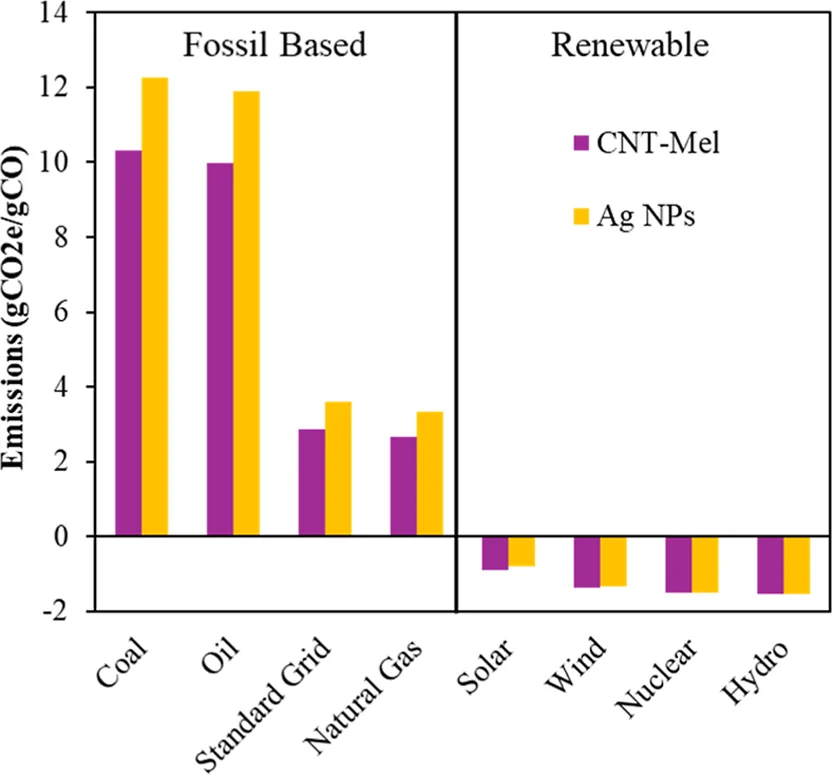
CO_2_e emissions for carbon capture and utilization using CNT-Mel and Ag NPs as electrocatalysts powered by different energy sources.

Figure 6 details the influence of different electricity sources on the emissions of a 100,000 kg per day CCU system under various energy source scenarios. Since electricity consumption dominates CCU system emissions, the carbon intensity of each energy source (Table S5) is a primary determinant of overall CO_2_e. As the carbon intensity of the energy source decreases, the emission differential between CNT-Mel and Ag NPs correspondingly narrows. Under a fully renewable electricity supply, the advantage of CNT-Mel becomes predominantly economic rather than environmental. A commercial CCU deployment using CNT-Mel and powered exclusively by hydro would yield the lowest new emissions, achieving a net removal of 1.53 gCO_2_e per gCO produced. However, because large-scale hydropower can impose substantial ecological and downstream impacts, nuclear- or wind-powered deployment may represent more environmentally balanced low-carbon options. Taken together, the TEA and LCA indicate that CNT-Mel enables superior economic performance and lower life cycle emissions than Ag NP cathodes across a wide range of realistic electricity scenarios.

## 5 Conclusion

In this study, up to 75 g per batch of a Ni/Fe-based M–N–C catalyst (CNT-Mel) was successfully synthesized by a repeatable and scalable moderate-temperature pyrolysis process using commercial CNTs as the catalyst feedstock. Material characterization (XPS, XRD, and TEM) confirmed the repeatability and scalability of the synthesis method, as all batch sizes produced catalysts with nearly identical properties. Highly competitive electrochemical performance was achieved consistently among all batch sizes with selectivity for CO above 98% at 500 mA cm^–2^ and mild onset overpotentials of –0.42 V. The industrial-grade CNTs have demonstrated similar electrochemical performance but are less expensive than the regular-grade CNTs. TEA results reveal a minimum selling price for CO when selling byproducts of 145 $/ton in the base case, $255 below the market price for CO. LCA results show that in the base case, operating a commercial eCO_2_R facility with the CNT-Mel catalyst will lead to 2.88 gCO_2_e/gCO emissions, which is 20% less than the benchmark Ag nanoparticle catalyst. The combined economic and emissions analysis indicates that reducing catalyst material cost or synthesis emissions has a relatively minor impact on the commercial viability and carbon footprint of CCU systems, whereas preserving high electrochemical performance at large-batch scales of syntheses is the dominant determinant of successful deployment. With that, CNT-Mel is superior to Ag NPs because of its superb CO selectivity and lower cell voltage requirement, besides its lower price tag, because CNT-Mel is composed of earth-abundant materials. Future research will focus on implementing large-batch CNT-Mel in larger, industrially relevant electrolyzers and evaluating long-term operational stability.

## Supplementary Material



## Notes

System boundary for TEA and LCA; TEM, HR-TEM, and HAADF-STEM images of indCNT-Mel-75g, Raw CNTs, and Raw indCNTs; cyclic voltammetry results used for ECSA calculations; surface elemental composition from XPS analyses; MEA testing results for CNT-Mel-75g; assumptions and specifications for TEA and LCA calculations; and example calculation flows for TEA and LCA (PDF)
