# Supplementary material for: Large-Scale Synthesis (75 g/Batch) of Single-Atom Catalysts for Selective Electrochemical CO2 Reduction to CO and Commercialization Potential Analysis
Source: ACS Omega. 2026 Jun 2;11(23):34554–67. doi: 10.1021/acsomega.6c02693 (PMC13750460; doi:10.1021/acsomega.6c02693)
Supplement: Supplementary file 1 [file ao6c02693_si_001.pdf]

Supporting Information

Large-Scale Synthesis (75 g/batch) of Single-Atom Catalysts for Selective Electrochemical CO_2 Reduction to CO and Commercialization Potential Analysis

Carter Racine[1], Ahmed Badreldin[1,2], John Pellessier[1], Yayun Chen[3], Shaoqin Chen[4], Shengyao Wang[1], Jin Feng[1], Chengcheng Fei[3], Yun Hang Hu[4], Ying Li[1,5*]

[1]J. Mike Walker '66 Department of Mechanical Engineering, Texas A&M University, College Station, TX 77843, USA

[2]Department of Chemical Engineering, University of Mississippi, Oxford, MS 38677, USA

[3]Department of Agriculture Economics, Texas A&M University, College Station, TX 77843, USA

[4]Department of Materials Science and Engineering, Michigan Technological University, Houghton, MI, 49931 USA

[5]Artie McFerrin Department of Chemical Engineering, Texas A&M University, College Station, TX 77843, USA

*Corresponding Author; email: yingli@tamu.edu

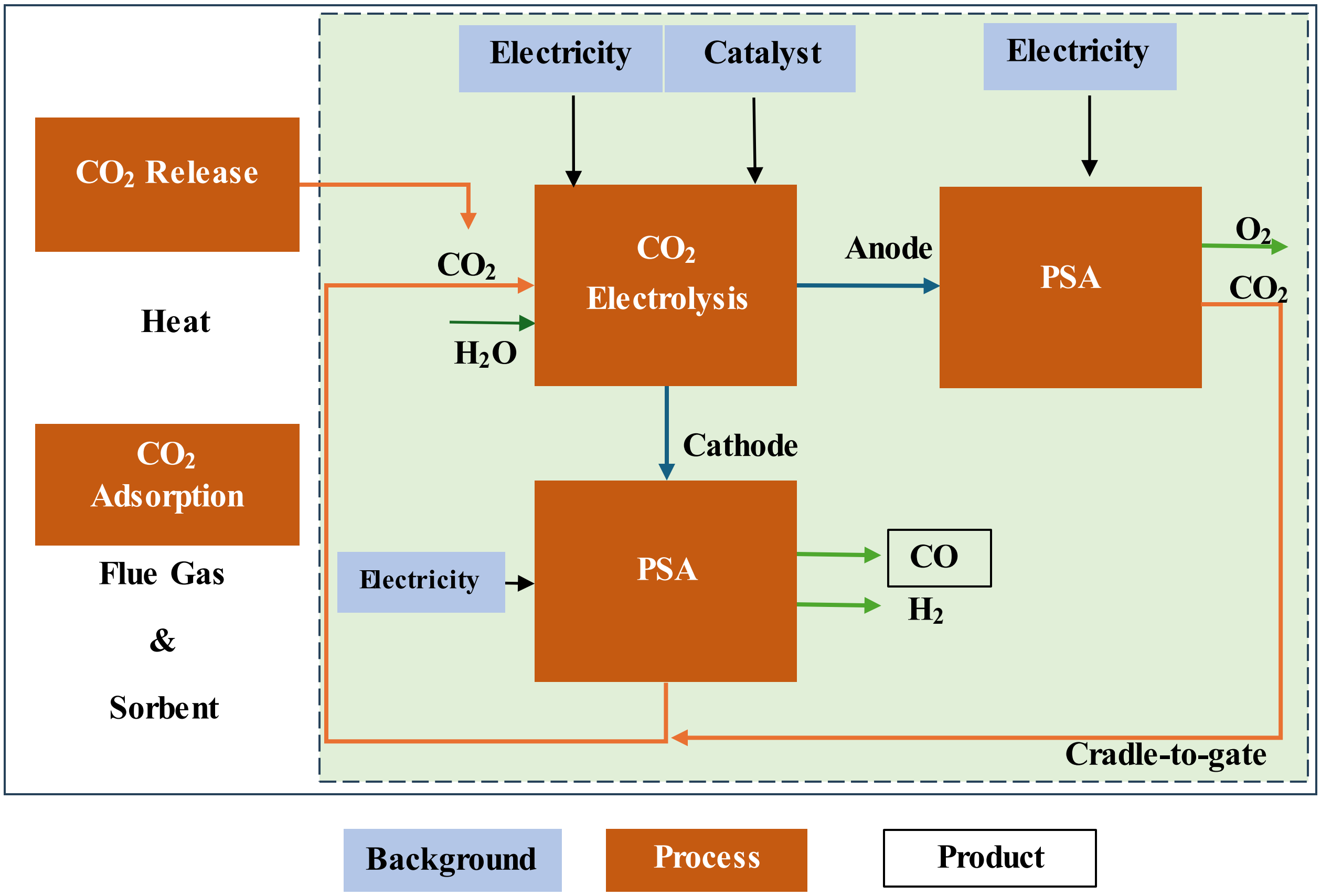

Figure S1. System Boundary for TEA and LCA

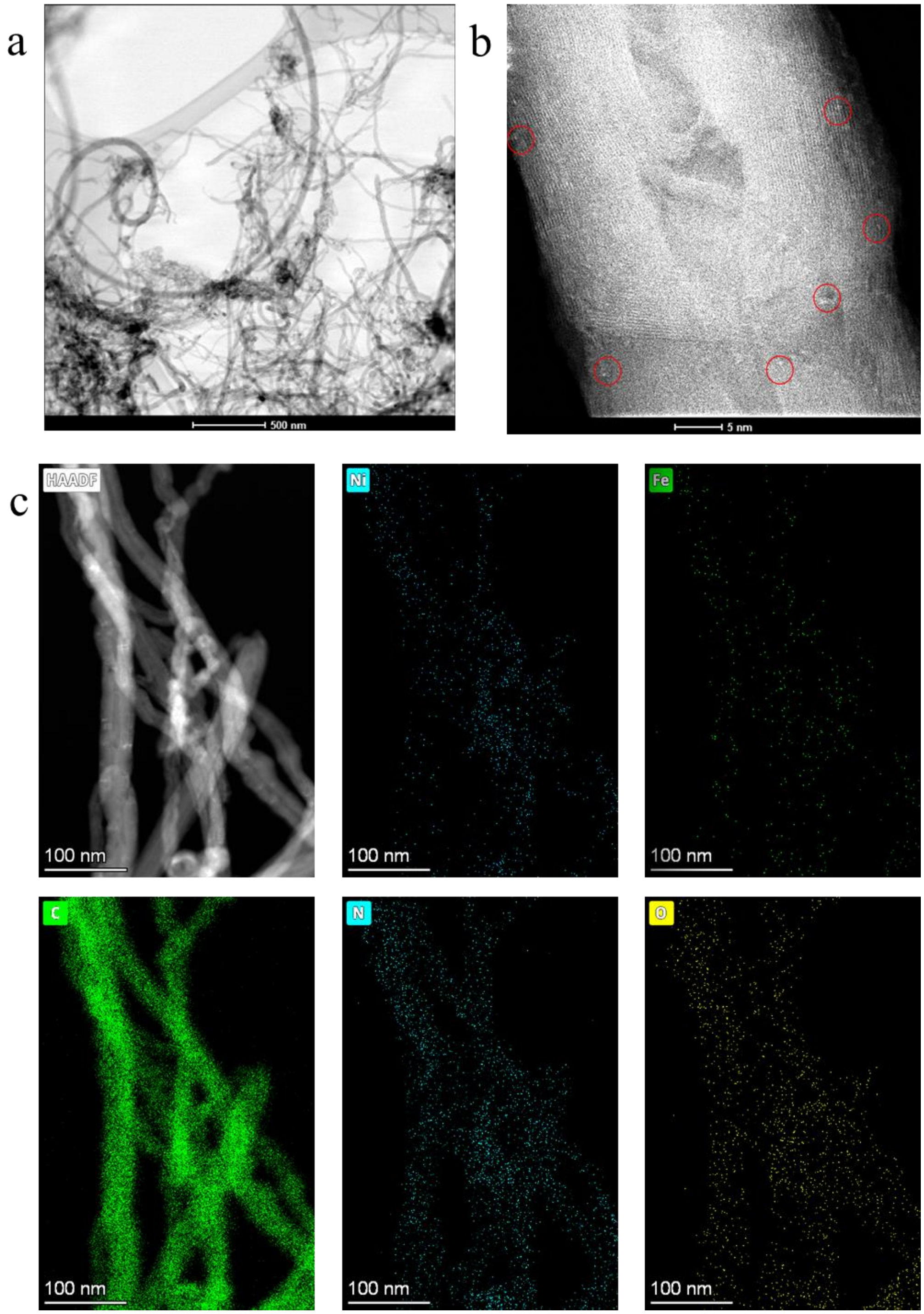

Figure S2. (a)TEM (b) High resolution TEM and (c) HAADF-STEM with corresponding elemental maps for indCNT-Mel-75g

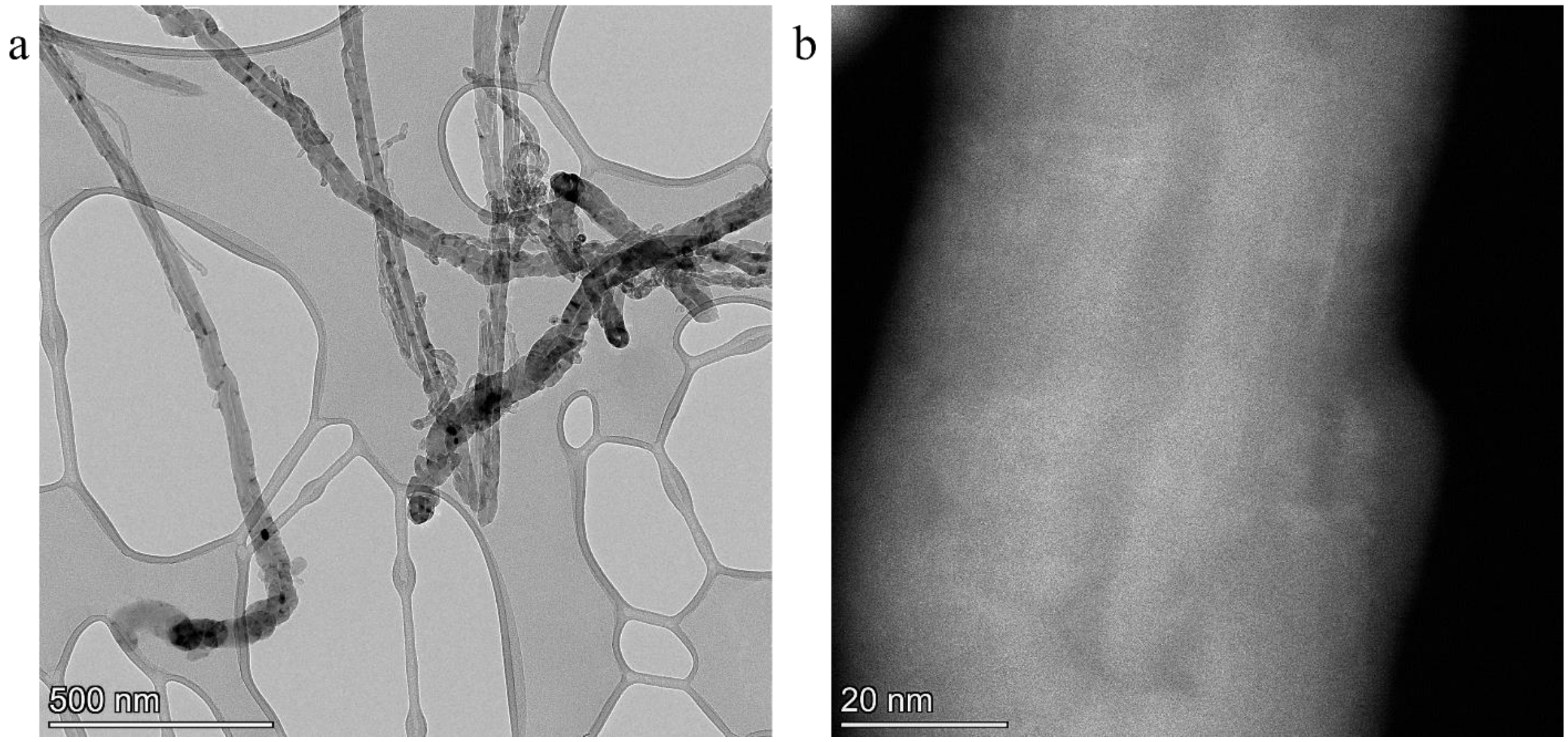

Figure S3. (a) TEM and (b) High Resolution TEM for Raw CNT

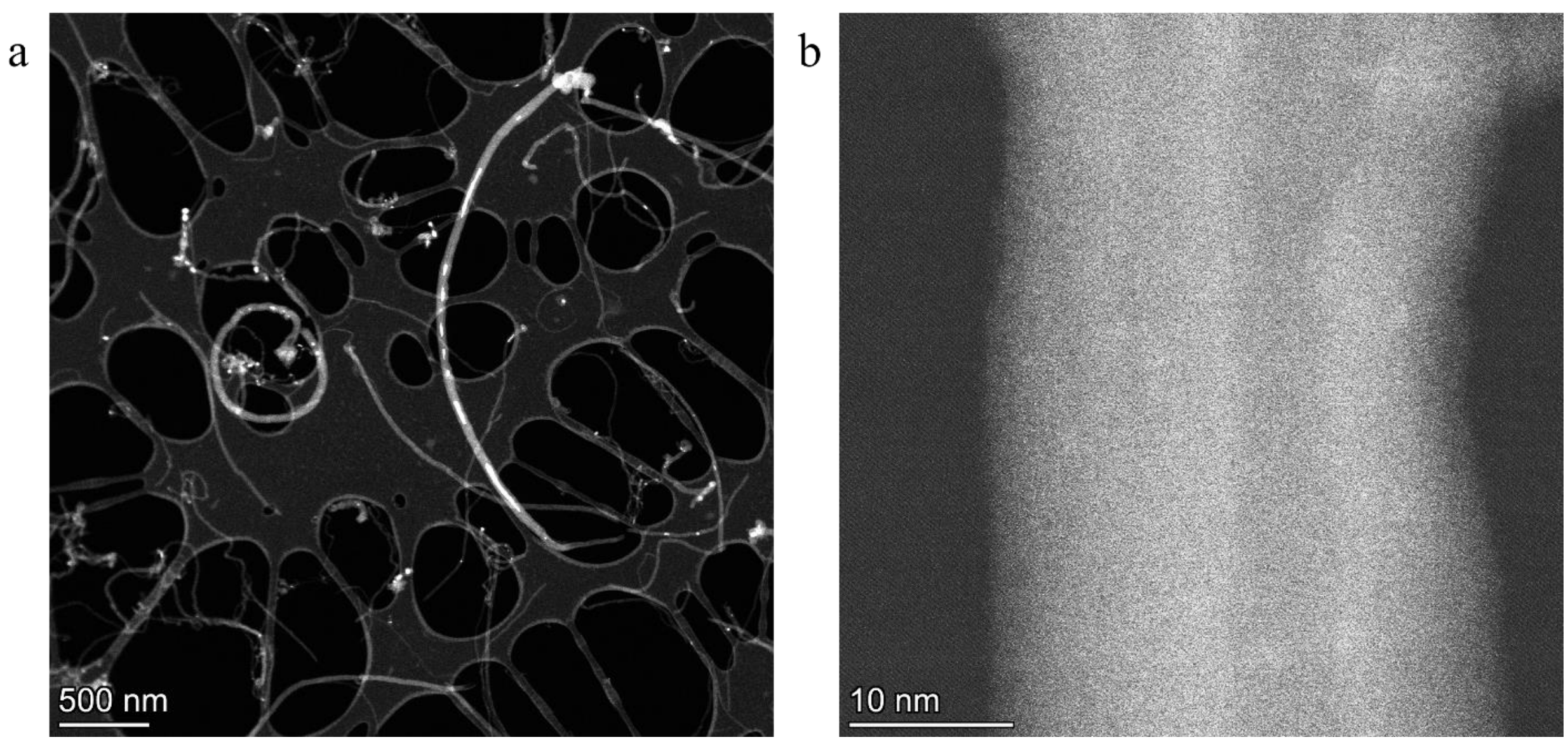

Figure S4. (a) TEM and (b) High Resolution TEM for Raw indCNT

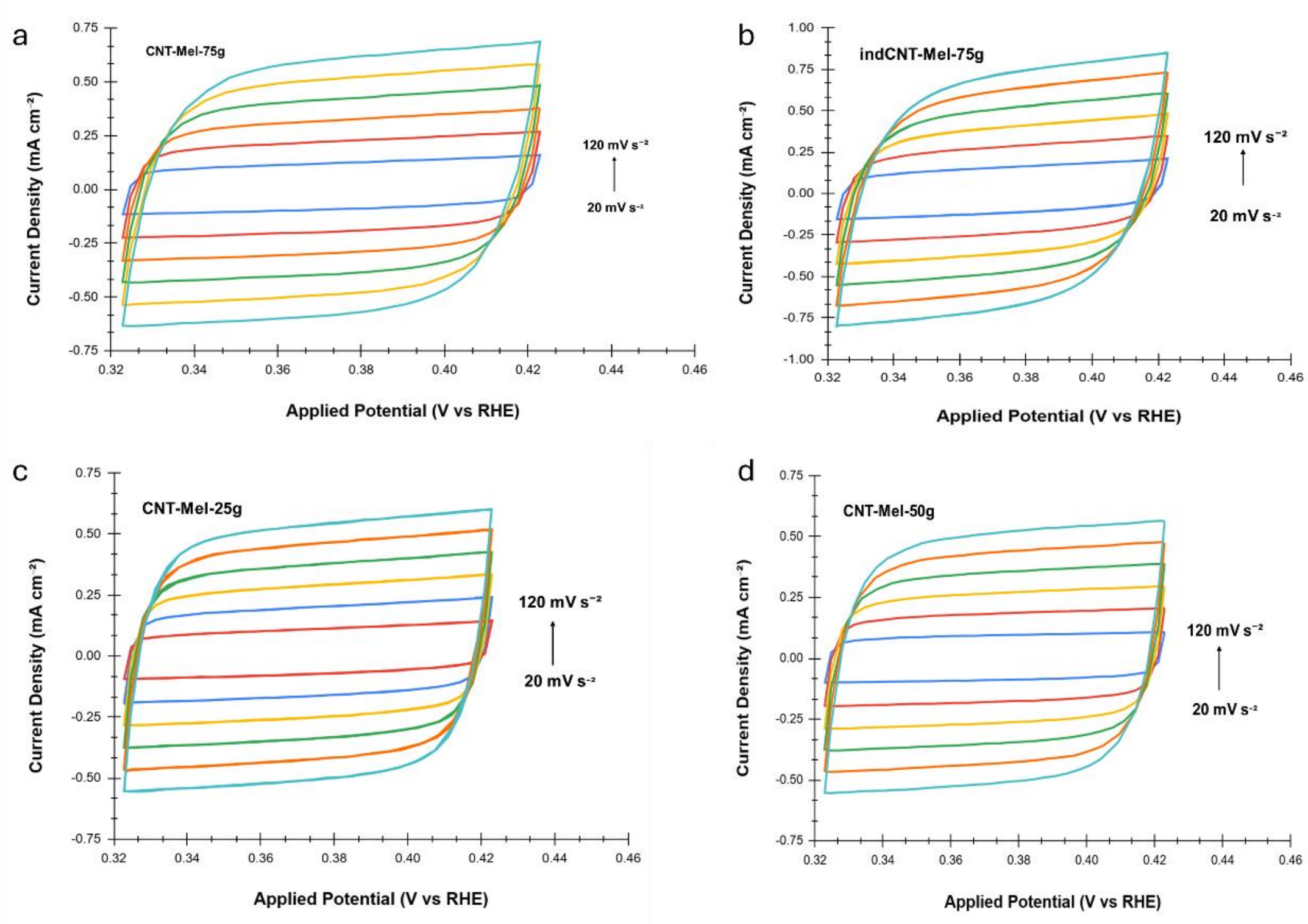

Figure S5. Cyclic voltammetry in a non-Faradaic window at different scan rates (20-120 mV s^{-2}) for (a) CNT-Mel-75g, (b) indCNT-Mel-75g, (c) CNT-Mel-25g, (d) CNT-Mel-50g

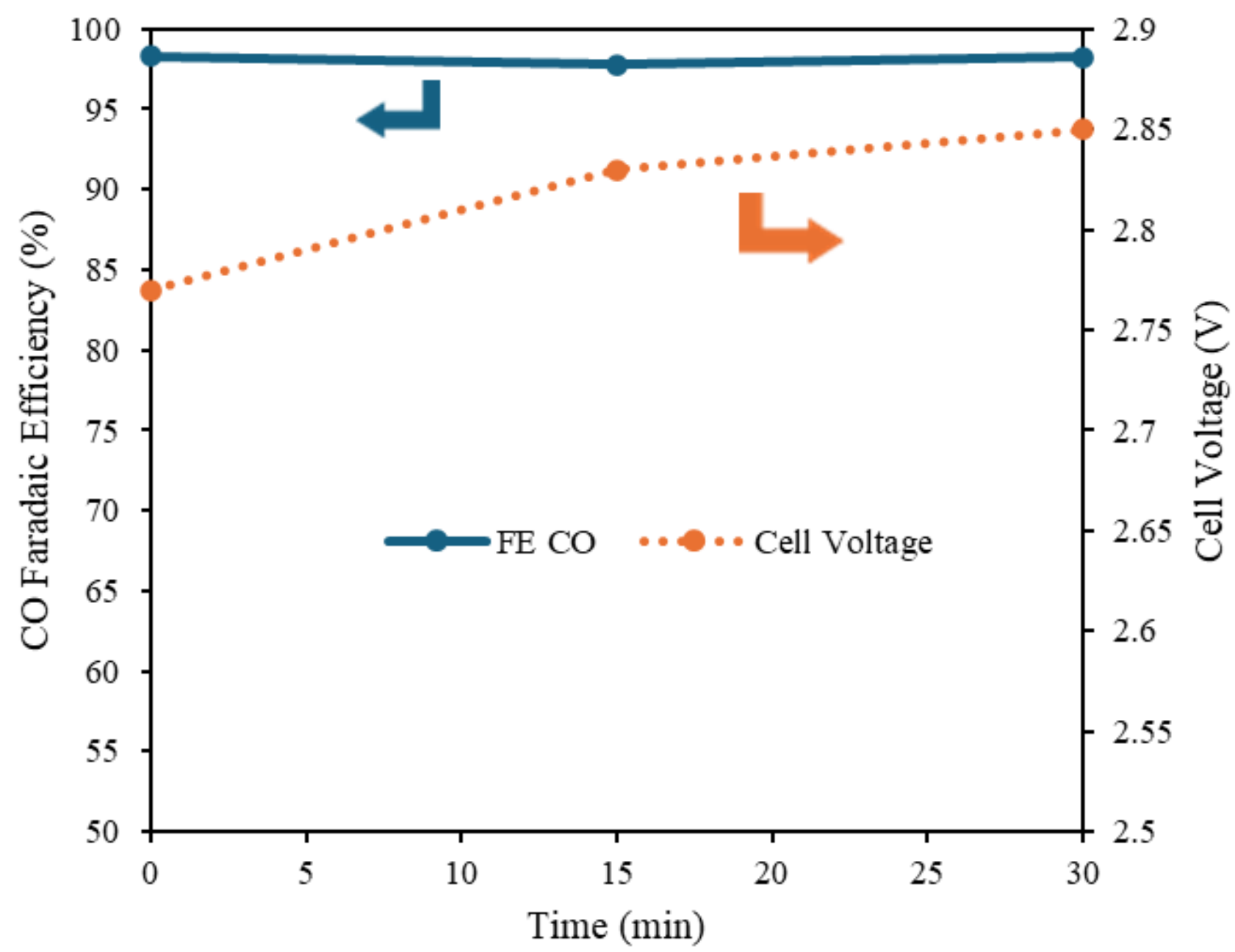

Figure S6. MEA testing result at 300 mA cm^{-2}

Table S1. Electrochemical Performance Metrics

	CNT-Mel			Ag NPs		
SPCE (%)	25 [1]	50 [1]	70 [1]	25 [1]	50 [1]	70 [1]
Carbonate Formation Rate (%)	50 [2]			50 [2]		
Current Density (mA cm^{-2})	300			300 [3]		
Applied Voltage (V)	2.8			3.4 [3]		
CO Product Selectivity (%)	98			91[3]		

Note: Current density, applied voltage and product selectivity values for CNT-Mel are derived experimentally, shown in Figure S6. There are three scenarios for SPCE: pessimistic 25%, base 50%, and optimistic 75%.

Table S2. System Assumption for TEA

**Parameter**	**System Assumption**
Production Rate (ton day^{-1})	100
Plant Lifetime (Years)	20
Operating Days Per Year (day $year^{-1}$)	350
LCOE (USD kwh^{-1})	0.03
Electrolyzer Cost Based on DOE H2A (USD m^{-2})[4]	919.669
Electrolyzer Maintenance Cost wrt Capital (%)	2.5
Capture Media	30% MEA
Capture Media Cost (USD ton^{-1})[5]	2454
Absorption Capacity (mol_{CO2} $molsolvent^{-1}$)[6]	0.45
Capture Efficiency (%)	90
Capture Capital Cost (USD kg^{-1})	0.000049
PSA Reference Cost (USD)	1989043
PSA Scaling Factor	0.7
PSA Reference Capacity (m^3 hr^{-1})	1000
PSA Electricity Operating Cost (kWh m^{-3})	0.25
Compressor Reference Cost (0.82 scaling factor)	5840
Distillation Reference Capacity (kg h^{-1})	77469
Distillation Reference Cost (USD)	15077002
Distillation Reference Operating Cost (USD day^{-1})	69455
Carbonate Formation (%)	50
Labor Cost wrt to Total CAPEX (%)	5
Electrolyzer Replacement Cost as % of Electrolyzer Capital Cost	15
Water Cost (USD gal^{-1})	0.0054
Compressor Work (kW)	49.3
Capture Energy Requirement (MJ/kg)[7]	3.8

Table S3. Financial assumptions for TEA

Parameter	Financial Assumption
Income Tax (%)	38.9
Return on Investment (%)	10
Market Price of Hydrogen (USD kg^{-1})	1.9
Market Price of Oxygen (USD kg^{-1})	0.4
Market Price of CO (USD kg^{-1})	0.4

Table S4. Atomic concentrations of Fe, Ni, and N for the catalyst samples from XPS analyses

Catalyst Sample	Fe Concentration (at.%)	Ni Concentration (at.%)	N Concentration (at.%)
CNT-Mel-25g	0.1	0.3	2.0
CNT-Mel-50g	0.2	0.3	2.7
CNT-Mel-75g	0.2	0.3	2.9
indCNT-Mel-75g	0.2	0.4	2.9
indRaw-CNT	<0.1	0.1	0
Raw-CNT	<0.1	<0.1	0

Table S5. Emission Factors

Input	Emission Factor
Melamine	17.8(gCO2e/g)[8]
CNTs	20 (gCO2e/g)[9]
Ag Nanoparticles	543(gCO2e/g) [10]
Argon Gas	2.81(gCO2e/g) [11]
Electricity: Standard Grid	434 (gCO_2e/kWh) [12]
Electricity: Coal	1,160 (gCO_2e/kWh) [12]
Electricity: Natural Gas	412 (gCO_2e/kWh) [12]
Electricity: Oil	1,128 (gCO_2e/kWh) [12]
Electricity: Nuclear	6.48 (gCO_2e/kWh) [12]
Electricity: Solar	21.2 (gCO_2e/kWh) [12]
Electricity: Wind	64.9 (gCO_2e/kWh) [12]
Electricity: Hydro	4.44 (gCO_2e/kWh) [12]

Supplementary Note 1: CNT-Mel-75g TEA Example Calculation

To show how TEA values were derived, a detailed sample calculation is shown below for the operation of the CNT-Mel-75g catalyst.

SN 1.1: Flowrates of Streams

This TEA models a plant with a production rate of 100,000 kg day^{-1} of cathodic CO. The required partial current density for the 2-electron CO reaction is:

Partial Current Needed: $100{,}000\frac{kg}{day} * \frac{day}{86{,}400s} * \frac{1000g}{kg} * \frac{mol}{28.01g} * 2e^- * 96{,}485\frac{C}{mol} = 7{,}973{,}756\ A$

To calculate total current factor in the 98% CO FE reached in Figure S6

Total Current Needed: $\frac{7{,}973{,}756\ A}{0.98}$= 8,136,486 A

Total electrolyzer area needed can then be calculated by dividing the total current by the cell specific current density of 300 mA cm^{-2}:

Electrolyzer Area: $\frac{8{,}136{,}486\ A}{0.300\ \frac{A}{cm^2}} * \frac{m^2}{10^4\,cm^2} = 2{,}712\ m^2$

The total electrolyzer area will allow the needed mass of catalyst per mass of CO produced to be calculated, using the catalyst loading and a replacement rate of once per year.

Catalyst Required per year: $2{,}712m^2 * 10^4\frac{cm^2}{m^2} * 1\frac{mg\ Cat}{cm^2} * \frac{1g}{1000mg} = 27{,}120g\ Cat$

To assist in LCA calculations, the amount of catalyst needed per gram of produced CO is also calculated.

gCO produced per year: $100{,}000\frac{kg\ CO}{day} * 350\ \frac{operating\ days}{year} * 1{,}000\frac{g}{kg} = 3.5 * 10^{10}gCO$

gCatalyst Required per gCO: $27{,}120g\ Cat\ \div\ 3.5 * 10^{10}gCO = 7.75 *\ 10^{-7}\ \frac{gCat}{gCO}$

Required power is then calculated by Power = Voltage * Current

Required Power: 2.8V * 8,136,486 A * $\frac{W}{10^6\,MW}$ = 22.78 MW

The flow rate of CO2 needed for this level of CO production is calculated with faradays law

CO_2 Flowrate Needed for Reaction: $\frac{7{,}973{,}756\ A*86{,}400\frac{s}{day}*0.044\frac{kgCO_2}{mol}}{2\frac{e^-}{mol\ CO_2} * 96{,}485\frac{C}{mol}} = 157{,}087\frac{kg}{day}$

Due to loss of CO_2 from carbonate formation and a lower than 100% SPCE, the actual CO_2 flowrate that must be provided is much higher. First, a carbonate formation (CF) rate of 50% is assumed, meaning that half of all the CO_2 provided to the cell will dissolve into the electrolyte forming carbonate, which will cross the AEM and be re-oxidized into CO_2 at the anode. Also, of the 50% CO_2 that remains gaseous, 50% of that will be left unreacted and flow out of the cathode outlet, due to the base case SPCE of 50% assumed in this sample calculation.

CO_2 Flow Rate Needed for Cell Operation: $\frac{157{,}087\ \frac{kg}{day}}{0.5(SPCE)*0.5(CF)} = 628{,}348\ \frac{kg}{day}$

Unreacted CO_2 exiting the cell must be separated from the other cathodic products using a pressure swing absorber (PSA). Therefore, a volumetric flow rate of unreacted CO_2 is needed, as the PSA power requirement is calculated using volumetric flow rates.

CO_2 Flow Rate at Cathode Outlet (Volumetric): $\frac{628{,}348\ \frac{kg}{day} * (1-0.5(CF)) * (1-0.5(SPCE))}{1.98 \frac{kg\ CO_2}{m^3} * 24 \frac{hrs}{day}} = 3{,}306\ \frac{m^3}{hr}$

Volumetric flow rate is needed for cathodic products CO (FE 98%) and H_2 (FE 2%) as well to calculate required PSA power. Faradays law is used to make these calculations.

CO Flow Rate at Cathodic Outlet (Volumetric): $\frac{8{,}136{,}486\ A*0.98}{2e^- * 96{,}485 \frac{C}{mol}} * \frac{0.028 \frac{kg}{mol}}{1.14 \frac{kg}{m^3}} * 3{,}600 \frac{s}{hr} = 3{,}655\ \frac{m^3}{hr}$

H_2 Flow Rate at Cathodic Outlet (Volumetric): $\frac{8{,}136{,}486\ \mathrm{A}*(1-0.98)}{2e^- * 96{,}485 \frac{C}{mol}} * \frac{0.002 \frac{kg}{mol}}{0.0899 \frac{kg}{m^3}} * 3{,}600 \frac{s}{hr} = 68\ \frac{m^3}{hr}$

The total gaseous flow rate out of the cathode is a sum of these three gases

Total Cathodic Outlet Flow Rate: $(3{,}306 + 3{,}655 + 68) \frac{m^3}{hr} = 7{,}028 \frac{m^3}{hr}$

The water flow rate required to perform the cathodic HER is:

Required Water for HER: $\frac{8{,}136{,}486\ \mathrm{A}*(1-0.98)}{2e^- * 96{,}485 \frac{C}{mol}} * 0.018 \frac{kg}{mol} * 0.2642 \frac{gal}{m^3} * 86{,}400 \frac{s}{day} = 346\ \frac{gal}{day}$

Volumetric flow rate is also needed for anodic output gases (CO_2 and O_2). It is assumed that the anodic oxygen evolution reaction (OER) operates at 100% FE, leading to substantial byproduct O_2 being produced in this 4-electron process. Carbonate formed at a rate of 50% by CO_2 and alkaline catholyte crosses over the AEM and is oxidized back into CO_2, leading to substantial CO_2 flow out of the anode which must be recycled.

CO_2 Flow Rate at Anodic Outlet (Volumetric): $\frac{628{,}348\ \frac{kg}{day} * (1-0.5(CF))}{1.98 \frac{kg\ CO2}{m^3} * 24 \frac{hr}{day}} = 6{,}611\ \frac{m^3}{hr}$

O_2 Flow Rate at Anodic Outlet (Volumetric): $\frac{8{,}136{,}486\ \mathrm{A}}{4e^- * 96{,}485 \frac{C}{mol}} * \frac{0.032 \frac{kg}{mol}}{1.429 \frac{kg}{m^3}} * 3600 \frac{s}{hr} = 1{,}700 \frac{m^3}{hr}$

The total anodic gas flowrate is $6{,}611\ \frac{m^3}{hr} + 1{,}700 \frac{m^3}{hr} = 8{,}311 \frac{m^3}{hr}$

The water needed for anodic OER is:

Needed Water for OER: $\frac{8{,}136{,}486\ \mathrm{A}}{4e^- * 96{,}485 \frac{C}{mol}} * 0.018 \frac{kg}{mol} * 0.2642 \frac{gal}{kg} * 86{,}400 \frac{s}{day} = 8{,}482\ \frac{gal}{day}$

SN 1.2: CAPEX Calculations

CAPEX calculations will involve the cost of the electrolyzer, separation units for gas (PSA), compressors, amine-based absorption and stripping towers, and the balance of plant (BoP). The referenced DOE H2A electrolyzer cost is 919.67 USD/m^2, therefore the electrolyzer capital cost is:

Electrolyzer Cost: $919.67 \frac{USD}{m^2} * 2{,}712 m^2 = 2{,}494{,}291\ USD$

Following the H2A model, the BoP for the electrolyzer is 35% of the total cost, with the balance being for the stack.

BoP Capital Cost: 2,494,145 USD * (0.35/0.65) = 1,343,080 USD.

With a levelized capture capital cost of 0.000049 USD kg^{-1}, therefore multiplying this levelized cost by the needed CO_2 flow rate will provide the CO_2 capture capital cost. In SN1.1 the *CO_2 Flow Rate Needed for Cell Operation* was calculated by addressing the impact of carbonate loss and SPCE on the *CO_2 Flowrate Needed for Reaction*. However, because of the implementation of anodic and cathodic PSAs, the CO_2 left unreacted can be recycled back into the feed stream. Therefore, the CO_2 capture system must only replace what is lost to the eCO_2RR reaction.

CO_2 Capture Cost: $157{,}087\,\frac{kg}{day} * 0.000049\frac{USD}{kg} * 350\frac{days}{yr} * 20yrs = 53{,}881$ USD

There will need to be three separate PSAs for this system, two at the cathode output and one at the anode output. The first PSA on the cathode side will separate CO_2 from H_2 and CO, then the second will separate H_2 and CO. The anode PSA will separate CO_2 and O_2. This is calculated by multiplying the PSA reference cost by the sum of gas flow rates entering each PSA divided by the PSA reference capacity and raised to the PSA scaling factor.

PSA Capital Cost: $\left(\frac{8311\frac{m^3}{hr}}{1000\frac{m^3}{hr}}^{0.7} + \frac{7028\frac{m^3}{hr}}{1000\frac{m^3}{hr}}^{0.7} + \frac{(7028-3306)\frac{m^3}{hr}}{1000\frac{m^3}{hr}}^{0.7}\right) * 1{,}989{,}043\ USD = 21{,}537{,}547\ USD$

Compressor capital cost can be calculated using the Douglas relation, assuming a compressor efficiency of 82% and a compressor reference cost of 5,840 USD. Based on an inlet flow rate of 628,348 kg/day of CO_2 (13,223 m^3/hr), 49.3 kW of work is needed for the compressor.

Compressor Capital Cost: $5{,}840\ USD * (49.3kW^{0.82}) = 142{,}741\ USD$

Total Capital Cost: 25,571,538 USD

SN1.3: OPEX Calculations

Electrolyzer operation costs are calculated using a LCOE of 0.03 USD kWh^{-1}.

Electrolyzer Operating Cost: $22.78MW * \frac{1000kW}{1MW} * \frac{0.03\ USD}{kWh} * \frac{24hrs}{day} = 16403\ \frac{USD}{day}$

Electrolyzer maintenance costs are based on the H2A model, where a maintenance cost of 2.5% of capital cost is assumed.

Electrolyzer Maintenance Cost: $2{,}494{,}291\ USD * \frac{year}{350\ days} * 0.025 = 178\ \frac{USD}{day}$

Electrolyzer replacement costs are taken as 15% of capital cost and are expected to be implemented every 5 years.

Replacement Cost: $2{,}494{,}291\ USD * \frac{year}{350\ days} * \frac{0.15}{5\ years} = 214\ \frac{USD}{day}$

PSA operating costs are calculated using the anodic and cathodic exit flowrates multiplied by the 0.25 kWh m^{-3} scaling factor.

PSA Operating Cost: $0.25\frac{kWh}{m^3} * \left(8311\frac{m^3}{hr} + 7028\frac{m^3}{hr} + (7028-3306)\frac{m^3}{hr}\right) * 0.03\frac{USD}{kWh} * 24\frac{hrs}{day} = 3431\frac{USD}{day}$

CO_2 capture operating cost can be calculated based on referenced energy requirements from Table S1.

CO_2 Capture Energy Requirement: $157{,}087\ \frac{kg}{day} * 3.8 \frac{MJ}{kg} = 596{,}931\ \frac{MJ}{day}$

CO_2 Capture Operating Cost: $596{,}931\ \frac{MJ}{day} * \frac{1\ kWh}{3.6\ MJ} * 0.03 \frac{USD}{kWh} = 4{,}975\ \frac{USD}{day}$

CO_2 compression operating cost is then calculated using 0.35 GJ ton^{-1}

CO_2 Compression Energy Requirement: $628{,}348\ \frac{kg}{day} * 0.35 \frac{GJ}{ton} * \frac{ton}{10^3\ kg} = 220\ \frac{GJ}{day}$

CO_2 Compression Operating Cost: $220\ \frac{GJ}{day} * \frac{1\ MWh}{3.6\ GJ} * \frac{10^3\ kWh}{MWh} * 0.03 \frac{USD}{kWh} = 1833 \frac{USD}{day}$

The upstream 30% MEA sorbent capture cost is then calculated

Upstream Sorbent Operating Cost: $157{,}087\ \frac{kg}{day} * \frac{mol\ CO_2}{0.044\ kg} * \frac{MEA}{0.45\ mol\ CO_2 * 0.98} * \frac{61.08 * 10^{-6} ton\ MEA}{mol\ MEA} * \frac{2425\ USD}{ton\ MEA} * \frac{year}{20 years * 350\ days} = 171\ \frac{USD}{day}$

Water operating cost is calculated from the sum of needed water for OER and HER.

Water Operating Cost: $0.0054 \frac{USD}{gal} * \left(8{,}482\ \frac{gal}{day} + 346\ \frac{gal}{day}\right) = 48 \frac{USD}{day}$

The labor cost is taken as 5% of the total CAPEX levelized per day.

Labor Cost: $\frac{25{,}733{,}180\ \text{USD}}{20 years\ * 350\ \frac{years}{day}} * 0.05 = 184 \frac{USD}{day}$

Therefore, total OPEX is 27,437 USD day^{-1}

Supplementary Note 2: CNT-Mel-75g LCA Example Calculation

To show how LCA values were derived, a detailed sample calculation is shown below for the synthesis and operation of the CNT-Mel-75g catalyst.

SN 2.1: Utilized CO_2

To calculate the amount of CO_2 utilized when producing CO electrochemically, a simple ratio of molecular weights is used. This is viable as CO_2 recycling is factored into the model therefore 100% total utilization is assumed.

Utilized CO_2: $\frac{44\frac{gCO_2}{mol}}{28\frac{gCO}{mol}} = 1.57\,\frac{gCO_2e}{gCO}$

SN 2.2: Catalyst Synthesis

Catalyst synthesis emissions come from three sources: catalyst feedstocks, electricity and Ar gas usage. Catalyst feedstock emissions are determined from the product of the mass ratio of feedstock to final catalyst, the emissions factor (Table S6) of the material and the needed catalyst per gram of CO produced (SN 1.1 *gCatalyst/ gCO*).

Melamine Emissions: $1\,\frac{\text{g Melamine}}{\text{gCNT\_Mel\_75g}} * 17.8\,\frac{\text{gCO}_2\text{e}}{\text{gMelamine}} * 7.75 * 10^{-7}\,\frac{\text{gCat}}{\text{gCO}} = 1.38 * 10^{-5}\,\frac{\text{gCO}_2\text{e}}{\text{gCO}}$

RawCNTs Emissions: $1\,\frac{\text{g RawCNT}}{\text{gCNT\_Mel\_75g}} * 20\,\frac{\text{gCO}_2\text{e}}{\text{gRawCNT}} * 7.75 * 10^{-7}\,\frac{\text{gCat}}{\text{gCO}} = 1.55 * 10^{-5}\,\frac{\text{gCO}_2\text{e}}{\text{gCO}}$

To calculate the emissions due to electricity consumption, the operating power is multiplied by the operating time, which is then multiplied by the emissions factor of standard electricity. Next this is divided by the amount of catalyst per batch and finally multiplied by the amount of catalyst needed per gram of CO produced.

Pyrolysis Electricity Emissions: $\frac{1.2kW * 3hrs * 434\frac{gCO_2e}{kWh}}{75\ gCat} * 7.75 * 10^{-7}\,\frac{\text{gCat}}{\text{gCO}} = 1.61*10^{-5}\,\frac{\text{gCO}_2\text{e}}{\text{gCO}}$

To calculate the material emissions from the Ar gas utilized in the pyrolysis, the operating time is multiplied by the flow rate and density of the gas and then divided by the amount of catalyst per batch to give the mass of Ar used in the synthesis. This is then multiplied by the emissions factor for Ar and the amount of catalyst needed to produce 1 g of CO to give the emissions due to Ar gas utilization.

Argon Mass Utilization: $\frac{3hrs*60\frac{min}{hr}*50sccm*0.00178 4\frac{g}{cm^3}}{75g} = 0.214\,\frac{\text{g Ar gas}}{\text{gCat}}$

Argon Emissions: $0.214\,\frac{\text{g Ar gas}}{\text{gCat}} * 2.81\,\frac{\text{gCO}_2\text{e}}{\text{gAr gas}} * 7.75 * 10^{-5}\,\frac{\text{gCat}}{\text{gCO}} = 4.66*10^{-7}\,\frac{\text{gCO}_2\text{e}}{\text{gCO}}$

Total Synthesis Emissions: $(1.61*10^{-5} + 4.66*10^{-7} + 1.55*10^{-5} + 1.38*10^{-5})\,\frac{\text{gCO}_2\text{e}}{\text{gCO}} = 4.59*10^{-5}\,\frac{\text{gCO}_2\text{e}}{\text{gCO}}$

SN 2.4: Electrolysis

The emissions due to electricity consumed during electrolysis are calculated by dividing the plants' daily power usage (SN1.1 *Required Power*) by the daily mass of CO produced and multiplying this value by the emission factor for electricity.

Electrolysis Electricity Emissions: $\frac{22780\ kW*24\frac{hrs}{day}}{100000\frac{kgCO}{day}*1000\frac{g}{kg}} * 434\frac{gCO_2e}{kWh} = 2.37\ \frac{\mathrm{gCO_2e}}{\mathrm{gCO}}$

SN 2.5: Separation

Cathode Output

To calculate cathodic separation emissions the volumetric output of CO, CO_2 and H_2 must be calculated first. This is already done in SN 1.1, now the m^3/hr values need to converted m^3/gCO.

Carbon Monoxide Output: $3{,}655\ \frac{m^3}{hr} * \frac{24hr}{day} * \frac{1\ day}{100{,}000kgCO} * \frac{1kgCO}{1000gCO} = 0.000877\frac{m^3}{gCO}$

Carbon Dioxide Output: $3{,}306\ \frac{m^3}{hr} * \frac{24hr}{day} * \frac{1\ day}{100{,}000kgCO} * \frac{1kgCO}{1000gCO} = 0.000793\frac{m^3}{gCO}$

Hydrogen Output: $68\ \frac{m^3}{hr} * \frac{24hr}{day} * \frac{1\ day}{100{,}000kgCO} * \frac{1kgCO}{1000gCO} = 0.0000163\frac{m^3}{gCO}$

Next, the sum of all output gases is multiplied by the PSA electricity operating cost (Table S1) and then multiplied by the electricity emissions factor.

Cathodic Separation Emissions: $0.25\frac{kWh}{m^3} *(0.000877\ \frac{\mathrm{m^3}}{\mathrm{gCO}} + 0.000793\ \frac{m^3}{gCO} + 0.0000163\ \frac{m^3}{gCO}) * 434\frac{gCO_2e}{kWh}\ =\ 0.183\ \frac{\mathrm{gCO_2e}}{\mathrm{gCO}}$

Anode Output

The gas flowing out of the anode also must be separated, O_2 gas is produced at the anode (OER) and CO_2 from oxidized carbonate crossover is present. Therefore, a similar calculation as cathode output must be done for O_2 and crossed over CO_2 to find the PSA emissions from this side of the cell. The anodic OER is assumed to be operating at 100% FE O_2.

Oxygen Output: $1{,}700\frac{m^3}{hr} * \frac{24hr}{day} * \frac{1\ day}{100{,}000kgCO} * \frac{1kgCO}{1000gCO} = 0.000408\ \frac{m^3}{gCO}$

CO_2 Output: $6{,}611\ \frac{m^3}{hr} * \frac{24hr}{day} * \frac{1\ day}{100{,}000kgCO} * \frac{1kgCO}{1000gCO} = 0.00159\frac{m^3}{gCO}$

Anodic Separation: $0.25\frac{kWh}{m^3} *(0.000408\ \frac{\mathrm{m^3}}{\mathrm{gCO}} + 0.00159\ \frac{m^3}{gCO}) * 434\frac{gCO_2e}{kWh}\ =\ 0.216\ \frac{\mathrm{gCO_2e}}{\mathrm{gCO}}$

Total Separation Emissions: $0.183\ \frac{\mathrm{gCO_2e}}{\mathrm{gCO}} + 0.216\ \frac{\mathrm{gCO_2e}}{\mathrm{gCO}} = 0.399\ \frac{\mathrm{gCO_2e}}{\mathrm{gCO}}$

SN 2.7 CO_2 Capture Emissions

To calculate CO_2 capture emissions, the CO_2 capture energy requirement will be used from SN 1.3 *CO_2 Capture Energy Requirement*.

CO_2 Capture Emissions: $596{,}931\ \frac{MJ}{day} * \frac{kWh}{3.6MJ} * 434\frac{gCO_2e}{kWh} * \frac{day}{10^8\,gCO} = 0.720\frac{\mathrm{gCO_2e}}{\mathrm{gCO}}$

SN 2.8 CO_2 Compression Emissions

To calculate CO_2 compression emissions, the CO_2 compression energy requirement will be used from SN1.3 *CO_2 Compression Energy Requirement*.

CO_2 Compression Energy Requirement: $220\ \frac{GJ}{day} * \frac{10^3\,MJ}{GJ} * 434\frac{gCO_2e}{kWh} * \frac{day}{10^8\,gCO} = 0.954\ \frac{\mathrm{gCO_2e}}{\mathrm{gCO}}$

SN 2.7 Net Emissions

Summing all sources of emissions results in the net CO_2 emissions per gram of produced CO.

Net Emissions: $(-1.57 + 4.59 * 10^{-5} + 2.37 + 0.399 + 0.720 + 0.954)\ \frac{gCO_2e}{gCO} = 2.88\ \frac{gCO_2e}{gCO}$

References

(1) Badreldin, A.; Li, Y. A critical appraisal of advances in integrated CO2 capture and electrochemical conversion. *Chemical Science* **2025**, *16* (6), 2483–2513, 10.1039/D4SC06642A. DOI: 10.1039/D4SC06642A.

(2) Kim, J. Y. T.; Zhu, P.; Chen, F.-Y.; Wu, Z.-Y.; Cullen, D. A.; Wang, H. Recovering carbon losses in CO2 electrolysis using a solid electrolyte reactor. *Nature Catalysis* **2022**, *5* (4), 288–299. DOI: 10.1038/s41929-022-00763-w.

(3) Ko, Y.-J.; Lim, C.; Jin, J.; Kim, M. G.; Lee, J. Y.; Seong, T.-Y.; Lee, K.-Y.; Min, B. K.; Choi, J.-Y.; Noh, T.; et al. Extrinsic hydrophobicity-controlled silver nanoparticles as efficient and stable catalysts for CO2 electrolysis. *Nature Communications* **2024**, *15* (1), 3356. DOI: 10.1038/s41467-024-47490-3.

(4) Jouny, M.; Luc, W.; Jiao, F. General Techno-Economic Analysis of CO2 Electrolysis Systems. *Industrial & Engineering Chemistry Research* **2018**, *57* (6), 2165–2177. DOI: 10.1021/acs.iecr.7b03514.

(5) Davis, J.; Rochelle, G. Thermal degradation of monoethanolamine at stripper conditions. *Energy Procedia* **2009**, *1* (1), 327–333. DOI: https://doi.org/10.1016/j.egypro.2009.01.045.

(6) Chai, S. Y. W.; Ngu, L. H.; How, B. S. Review of carbon capture absorbents for CO2 utilization. *Greenhouse Gases: Science and Technology* **2022**, *12* (3), 394–427. DOI: https://doi.org/10.1002/ghg.2151.

(7) Luis, P. Use of monoethanolamine (MEA) for CO2 capture in a global scenario: Consequences and alternatives. *Desalination* **2016**, *380*, 93–99. DOI: https://doi.org/10.1016/j.desal.2015.08.004.

(8) Vujanović, A.; Puhar, J.; Krajnc, D.; Awad, P.; Čuček, L. Reducing the environmental impacts of the production of melamine etherified resin fibre. *Sustainable Production and Consumption* **2022**, *29*, 479–494. DOI: https://doi.org/10.1016/j.spc.2021.10.014.

(9) Teah, H. Y.; Sato, T.; Namiki, K.; Asaka, M.; Feng, K.; Noda, S. Life Cycle Greenhouse Gas Emissions of Long and Pure Carbon Nanotubes Synthesized via On-Substrate and Fluidized-Bed Chemical Vapor Deposition. *ACS Sustainable Chemistry & Engineering* **2020**, *8* (4), 1730–1740. DOI: 10.1021/acssuschemeng.9b04542.

(10) Pourzahedi, L.; Eckelman, M. J. Comparative life cycle assessment of silver nanoparticle synthesis routes. *Environmental Science: Nano* **2015**, *2* (4), 361–369, 10.1039/C5EN00075K. DOI: 10.1039/C5EN00075K.

(11) Gas Recovery & Recycle Ltd. *Improving Profitability and Reducing Carbon Footprint*. GR2L Gas Recycling Systems. https://gr2l.co.uk/co2-footprint-reduction/ (accessed 2025-12-19).

(12) Wernet, G.; Bauer, C.; Steubing, B.; Reinhard, J.; Moreno Ruiz, E.; Weidema, B. The ecoinvent database version 3 (Part I): Overview and methodology. *The International Journal of Life Cycle Assessment* **2016**, *21*, 1–13. DOI: 10.1007/s11367-016-1087-8.
